# Associating H_2_O_2-_and NO-related changes in the proteome of *Mycobacterium smegmatis* with enhanced survival in macrophage

**DOI:** 10.1038/s41426-018-0210-2

**Published:** 2018-12-13

**Authors:** Naadir Ganief, Jessica Sjouerman, Claudia Albeldas, Kehilwe C. Nakedi, Clemens Hermann, Bridget Calder, Jonathan M. Blackburn, Nelson C. Soares

**Affiliations:** 1 Division of Chemical & System Biology, Department of Integrative Biomedical Science, University of Cape Town, Cape Town, South Africa; 2 Institute of Infectious Disease & Molecular Medicine, University of Cape Town, Cape Town, South Africa

## Abstract

*Mycobacterium* manages to evade the host cell immune system, partially owing to its ability to survive redox stress after macrophage engulfment. Exposure to redox stress has been linked to later replication, persistence, and latent infection. In this work, mass spectrometry was used to elucidate the cell-wide changes that occur in response to sublethal doses of hydrogen peroxide and nitric oxide over time, with *Mycobacterium smegmatis* being used as a model organism. A total of 3135 proteins were confidently assigned, of which 1713, 1674, and 1713 were identified under NO, H_2_O_2_, and control conditions, respectively. Both treatment conditions resulted in changes of protein expression from the DosR regulon as well as those related to lipid metabolism. Complementary to the changes in the proteome, sublethal exposure to NO and H_2_O_2_ improved the survival of the bacteria after macrophage infection. Our data indicate that pre-exposure to sublethal doses of these redox stressors causes an alteration in the expression of proteins related to lipid metabolism, suggesting a link between altered lipid metabolism and enhanced survival in macrophages.

## Introduction

*Mycobacterium tuberculosis*, the causative agent of tuberculosis (TB), presents a major health-care problem worldwide, with a global incidence of ~100 per 100,000 people and as much as 1/3 of the world’s population carrying a latent TB infection that can later reactivate. The success of *M. tuberculosis* is partially owing to the pathogen’s ability to evade the host immune system, lying dormant until the host becomes immunocompromised. Understanding the mechanisms of mycobacterial immune system evasion is necessary to combat the disease, which grows increasingly more elusive as multidrug-resistant strains emerge.

Mycobacteria are well recognized for their remarkable ability to sense engulfment by macrophages and then survive the numerous and different forms of macrophage bactericidal attack^1^. After macrophage engulfment, a mycobacterium is exposed to antimicrobial redox stresses, including reactive oxygen species (ROS); hydrogen peroxide (H_2_O_2_); superoxide (O_2_^−^); hydroxyl radicals (OH), mainly produced by NADPH oxidase (the main generator of ROS inside host cells), and reactive nitrogen species (RNS), specifically nitric oxide (NO), which is generated mainly by inducible nitric oxide synthase (iNOS)^1^. The importance of ROS in controlling mycobacterial infection has been shown by the observation that children with defective NADPH oxidase (NOX2) suffer from chronic granulomatous disease, are susceptible to TB, and are likely to develop serious complications after vaccination with Bacillus Calmette–Guérin^2^. A body of evidence indicates that survival of the bacilli to early macrophage redox actions is essential for later mycobacterial replication, persistence, and ultimately the establishment of latent infection^1,3,4^. The role of NO during mycobacterial infection of human macrophages has until recently been unclear, partly as a result of the low amounts of NO produced by these cells. Increasingly, evidence suggests that iNOS and the production of RNS play a significant role during the mycobacterial infection of human macrophages^5^.

Mycobacteria have evolved complex redox-sensing pathways to monitor both the intra- and extracellular redox environment. In this context, ROS and NO can also act as signaling molecules, promoting recognition at the atomic level^6^. In mycobacteria, ROS and NO exposure modulate multiple redox-sensing pathways, including the SigH/RshA, DosR/S/T, MosR, and WhiB families, to maintain redox homeostasis^7–11^. Both ROS and RNS, in a dose-dependent manner, can act as signaling molecules that trigger certain responses that determine mycobacterial intracellular survival and growth. Whether these molecules act synergistically by interfering at independent points within the same signal network or whether they activate completely different signal pathways in mycobacteria is unknown. A 2-dimensional gel-based study indicated that little overlap was observed between the H_2_O_2_- and NO-induced responses in *M. tuberculosis* cell cultures when exposed to either stress^12^. In contrast, recent transcriptomic analyses showed that NO exposure initiated much the same transcriptional responses as H_2_O_2_. However, unlike H_2_O_2_ exposure, NO exposure induced dormancy-related genes and caused dose-dependent bacteriostatic activity without killing^13^. Although some compelling transcriptomic data^14^ exist regarding mycobacterium responses to different concentrations of ROS or RNS, equivalent data at the proteome level still remain scarce. In this context, when comparing the protein fold changes obtained to the mRNA fold changes, Aebersold et al.^15,16^ reported that global correlations between mRNA and protein regulation in *M. tuberculosis* were surprisingly low, with *R*^2^ ranging between 0.06 and 0.52; furthermore, Cortes et al.^17^ found that protein expression is delayed compared with expression at the level of the transcriptome. These findings suggest that protein levels are essentially decoupled from gene expression^13,15,16^. This argues strongly for the need to gain a detailed understanding of ROS- and RNS-induced mycobacterial responses at the proteome level. However, *M. tuberculosis* is a slow-growing pathogen that must be cultured in BSL3 containment facilities, with culture times measured in months; furthermore, strict biosafety considerations now require that all new experimental procedures on *M. tuberculosis* need to be first demonstrated on a nonpathogenic organism in a BSL2 laboratory. The fast-growing, nonpathogenic *M. smegmatis* is therefore often used by researchers in the TB field as a model organism on which a wider range of experiments can be conducted more rapidly, before continuing the studies with *M. tuberculosis*^18–20^. For example, previous work in our laboratory employed a proteomic approach to investigate the effects of sublethal doses of rifampicin and vitamin C on *M. smegmatis*^21,22^. As a complementary approach to the growing transcriptomic data of mycobacterial response to NO and H_2_O_2_, we have carried out a large-scale, label-free, mass spectrometry-based study to investigate proteome dynamics at three distinct time points of *M. smegmatis* growth in vitro after exposure to sublethal concentrations of NO and H_2_O_2_. In addition, informed by our proteomics findings, we investigated the effect of pre-exposure to sublethal NO and H_2_O_2_ on the survival of *M. smegmatis* to macrophage attack, which to the best of our knowledge, is the first time that prior phenotypic adaption to oxidative stress has been directly associated with increased mycobacterial survival within the macrophage.

## Results

### Determination of sublethal concentrations of diethylenetriamine/nitric oxide (DETA-NO) and H_2_O_2_

#### treatment with DETA-NO

The DETA/NO adduct breaks down in solution and exhibits a half-life of ca. 20 h at pH 7.4, with an NO release duration > 24 h, to give a low, quasi-steady-state concentration of NO^23^. A previous study in *M. tuberculosi*s by Voskuil et al.^13^ reported that exposure to 0.05 mM DETA-NO introduced no growth defects but resulted in subsequent specific NO-related transcriptomic perturbation. On the other hand, higher concentrations of DETA-NO-induced general oxidative stress responses. Our results confirm that in *M. smegmatis* 0.05 mM DETA-NO confers no noticeable growth defect (Supplementary Figure 2), and based on our finding as well as previous observations, 0.05 mM was selected as the sublethal concentration for the current study.

### Treatment with H_2_O_2_

**Fig. 1:**
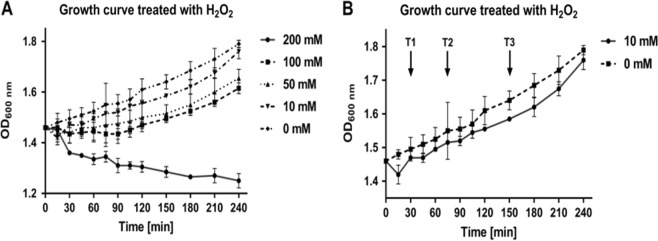
Growth curves for *Mycobacterium smegmatis* cultures treated with various H_2_O_2_ concentrations at mid-log phase. **a** The growth of *M. smegmatis* cultures as measured by absorbance at 600 nm when treated with increasing concentration of H_2_O_2_. Concentrations of 0–200 mM are represented. After treatment with 10 mM H_2_O_2_ cultures showed a slight growth defect, however growth recovered. Cultures treated with 50 mM and 100 mM H_2_O_2_ showed a larger growth defect when compared with cultures treated with 10 mM H_2_O_2_, and recovered growth much later. Cultures treated with 200 mM H_2_O_2_, showed an irreparable growth defect, suggesting in these conditions 200 mM H_2_O_2_ is lethal. **b** Arrows indicate the time points selected for further analysis at when treated with 10 mM H_2_O_2_

*Mycobacterium smegmatis* batch (shake flask) cultures at mid-log phase were treated with concentrations of H_2_O_2_ ranging from 10 mM to 200 mM (Fig. 1a). When treated with 200 mM H_2_O_2_, *M. smegmatis* culture suffered from an unrecoverable growth defect, whereas cultures treated with 50 and 100 mM H_2_O_2_ showed recovery of growth 120 min after treatment. Cultures treated with 10 mM H_2_O_2_ showed a growth defect within 30 min and had recovered to normal growth by 120 min. The sublethal dose selected for this study was therefore 10 mM H_2_O_2_. Based on growth curves of *M. smegmatis* treated with 10 mM H_2_O_2_, three time points after treatment were selected for cell harvesting and subsequent proteomic analysis (30, 75, and 150 min; T1, T2, and T3, respectively) (Fig. 1b). These three time points represent the onset of (T1), recovery from (T2), and post-recovery of (T3) H_2_O_2_ exposure. Note that cultures treated with DETA-NO (Supplementary Figure 2) were harvested at the same time points after treatment to maintain comparability between both conditions.

### Proteome dynamics

From a total of 1,357,381 spectra that were submitted, 797,173 were identified (58.7%). This resulted in the identification of 31,270 nonredundant peptides, of which 31,127 were unique to a protein group. The peptide identifications had a false discovery rate of 0.26%. From these peptides, a total of 3336 proteins were inferred, at a false discovery rate of 0.99%. For more details on the search engine parameters, see Supplementary Table 1. Protein groups were identified by at least one unique peptide, and at least two peptides were taken forward, which resulted in 3135 confident protein group assignments. To insure a reliable label-free quantification, we considered only those protein groups quantified in all experiments within a treatment for further analysis. After applying these filtering criteria, a total of 1713, 1674, and 1713 protein groups for the DETA-NO, H_2_O_2_ and untreated treatment groups, respectfully, remained. For details on the quantifiable proteins and their overlap between treatment conditions, see Supplementary Tables 2–5.

**Fig. 2:**
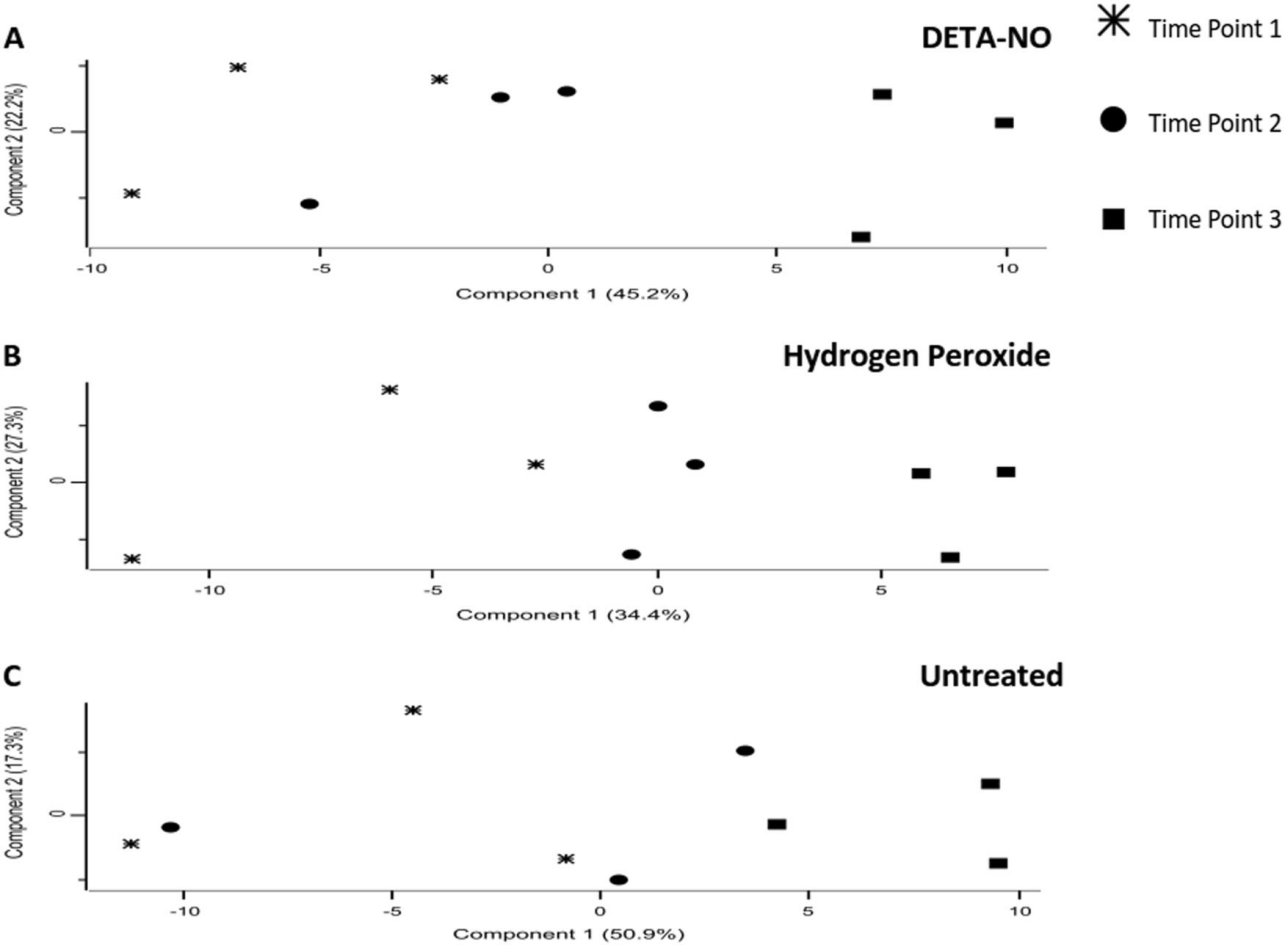
All replicates of all time points consisting of nine samples were used to generate a single plot for each treatment condition. Stars, circles, and squares represent samples from T1, T2, and T3, respectively. T1 and T2 are less distinct from each other in both treatment conditions (**a**—DETA-NO and **b**—hydrogen peroxide), and in the untreated condition there is little notable clustering of the first two time points (**c—**untreated). The treatment conditions samples from T3 are more distinct from the other time points. Untreated samples at T3 are notably less distinct from samples from T1 and T2. In the treated conditions, as the time of exposure increases, the proteomic signature becomes more distinct

To assess the reproducibility of the MaxQuant label-free quantification, we assessed the Pearson correlation between biological replicates. Multiscatter plots of each biological triplicate were then generated in Perseus^24^ (Supplementary Figure 3). The Pearson correlation scores for each comparison were >0.97, indicating a highly reproducible label-free quantitation. Principle component analysis (PCA) plots indicated that T3 of the treated samples (panel A and B) form a much more distinct cluster, whereas differences between the T1 and T2 samples seem to be less pronounced (Fig. 2). In contrast, PCA plots from the untreated group (panel C) did not cluster in a well-defined manner. In the case of the treated conditions, the relatively short time between sampling (45 min) may account for the increased similarity between the first and second time points, whereas the longer time between sampling for the second and third time points (75 min) may explain why the third time points form a more distinct cluster. In the case of the untreated condition, the absence of an introduced stress to drive the bacteria toward a distinct phenotype may account for the lack of clustering observed.

### Statistical analysis

Subsequent label-free differential protein abundance analysis was carried out, and proteins with a fold change of one standard deviation greater or less than the median fold change were considered for a *t* test (see Table 1 for more details). The fold change cutoff reduced the protein group list further to between 156 and 354 proteins per comparison (for a full list see Supplementary Tables 6–11). Significant differences were assessed using a two-tailed *t* test, and a *p* value of <0.05 was considered significantly different. For full details, see Supplementary Tables 12 and 13.

**Table 1:** The median fold changes for all comparisons as well as the standard deviation of the fold change

Time point comparison	Median fold change	Fold change standard deviation	Fold change cutoff(−) median − SD	Fold change cutoff( + ) median + SD
Untreated				
1 vs 2	0.005	0.154	−0.148	0.159
2 vs 3	0.008	0.297	−0.290	0.305
DETA-NO				
1 vs 2	0.003	0.167	−0.164	0.171
2 vs 3	0.009	0.283	−0.273	0.292
Hydrogen peroxide				
1 vs 2	−0.011	0.213	−0.224	0.202
2 vs 3	0.015	0.227	−0.212	0.243

The standard deviation was used to perform a fold change cutoff around the median fold change. Such that any fold change less than the fold change cutoff(−) was accepted as well as any fold change greater than the fold change cutoff( + ).

### Bioinformatics

#### Differentially expressed proteins unique to DETA-NO or H_2_O_2_ treatment

**Fig. 3:**
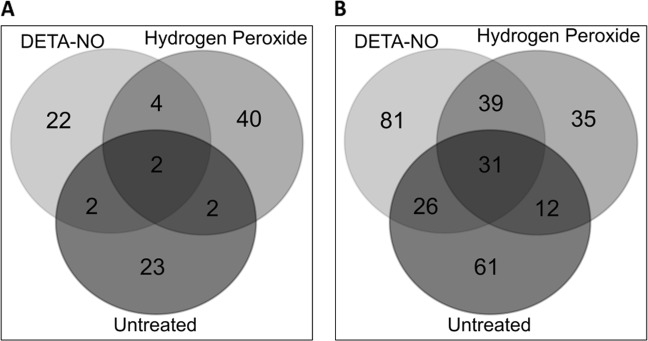
Proteins from each treatment condition that were found to have a differential abundance, as well as the overlap of these proteins between all conditions **a** 30, 48, and 29 proteins from DETA-NO, hydrogen peroxide, and untreated, respectively, were assessed for the overlap in protein identity. Of these, 22, 40, and 23 proteins were found to be unique to DETA-NO, H_2_O_2_, and untreated conditions, respectively. **b** 177, 117, and 130 proteins belonging to the DETA-NO, H_2_O_2_, and untreated conditions were assessed for overlap. It was found that 81, 35, and 61 proteins were unique to DETA-NO, H_2_O_2_, and untreated conditions, respectively

Venn diagrams were generated to identify proteins with differential expression that was unique to each treatment. When we compare T1 and T2, of the 95 proteins with differential abundance, 22, 40, and 23 proteins were uniquely different in the DETA-NO, H_2_O_2_, and control conditions, respectively (see Fig. 3 and Supplementary Table 15). In the comparison between T2 and T3, of the 281 proteins found to have a differential abundance, 81, 35, and 61 proteins were uniquely different in the DETA-NO, H_2_O_2,_ and control conditions, respectively. Notably, between T2 and T3, 39 proteins showed a change in abundance in both the DETA-NO and H_2_O_2_ treatment but not in the untreated cultures (see Fig. 3 and Supplementary Table 16 for full details).

### Changes in *M. smegmatis* proteome induced by exposure to sublethal concentration of DETA-NO

**Fig. 4:**
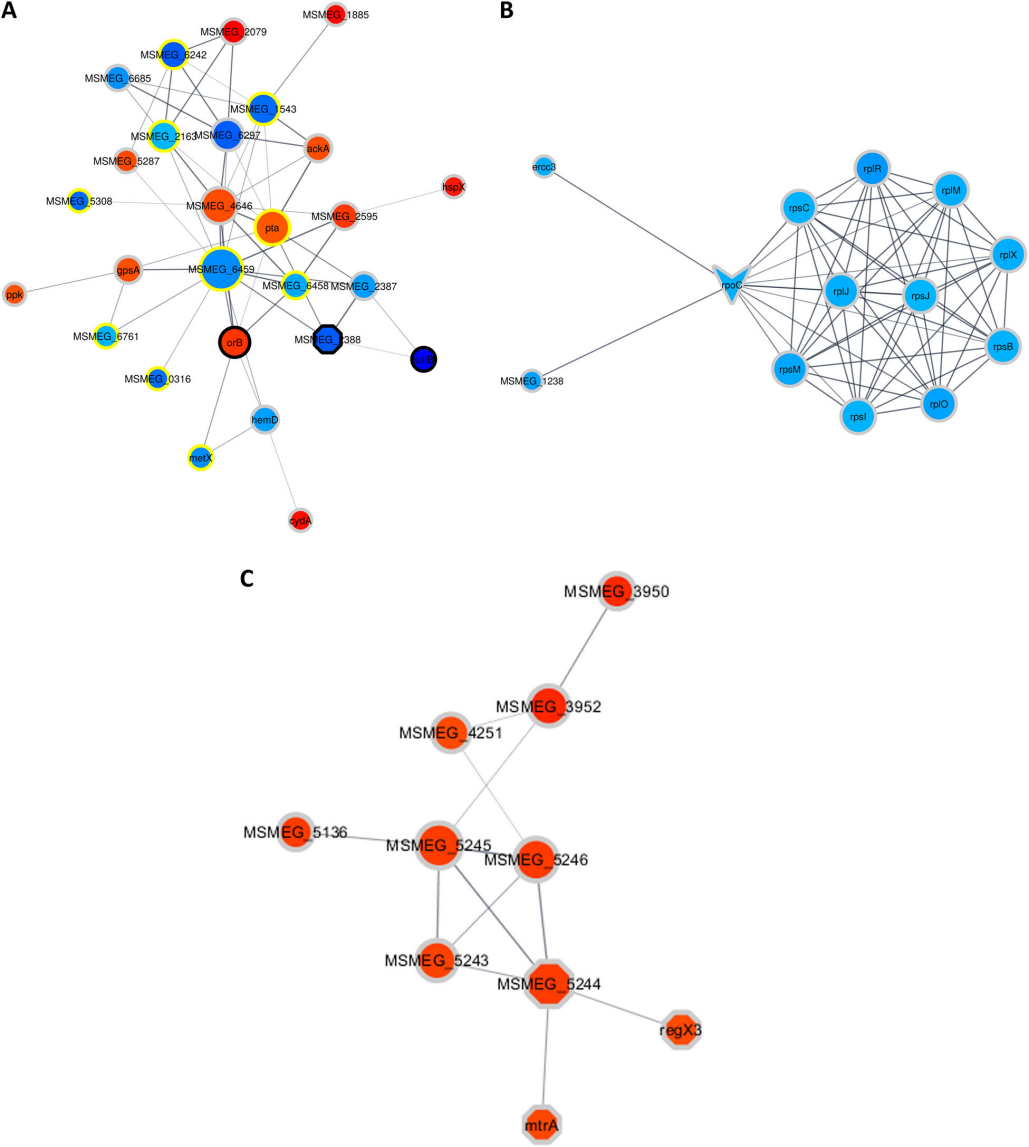
Clusters of associated proteins found to have significant differences in abundances as a result of DETA-NO pre-exposure (as determined by String-db) Clusters were determined by the EAGLE algorithm using ClusterVis via Cytoscape. The shape of the nodes denotes if a protein is a known drug target or virulence factor. Octagons represent known virulence factors, arrowheads represent known drug targets, and ellipses represent proteins that are not known to be either. The color of the nodes denotes the protein expression relative to the previous time point on a gradient of dark blue to deep red, with dark blue indicating the relatively lowest expression and deep red indicating the relatively highest expression. The color of the ring surrounding the nodes denotes when the protein showed differential expression, with black rings indicating that in both comparisons (T1 and T3) the protein showed altered expression, the yellow rings indicate altered expression in only the first comparison (T1), and the gray rings indicate altered expression in only the second comparison (T3). **a** shows proteins associated with carbohydrate metabolism involving pathways such as glycolysis/gluconeogenesis and the citrate cycle. **b** shows ribosomal proteins and some proteins associated with gene expression, most of which are downregulated. **c** shows the DosR regulon proteins

To assess the potential biological implications of the changes seen at the proteome level, we performed more downstream bioinformatics analyses. These included String-db and KEGG pathway mapper, and they revealed that, after DETA-NO treatment from T1 to T2, changes in the proteome were mainly related to acetyl-COA metabolism, including the increased levels of glucose transporters MSMEG_2116 and MSMEG_2117. In addition, the increased abundance of orB and MSMEG_4646 from T2 to T3 suggests a plausible increase in acetyl-CoA synthesis from both pyruvate (a product of glycolysis) and 2-oxoglutarate Figs. 3a and 4a. In addition, during T2 to T3, differential levels of proteins involved in glycolysis were observed, e.g., Pgm (up), MSMEG_4646 (up), MSMEG_2597 (up), MSMEG_1543 (down), adhE1 (up), and MSMEG_5287 (up) (Fig. 4a and Supplementary Figure 4A). These results point toward a possible increased synthesis of acetyl-CoA at T3. Within this context, lipid metabolism is intimately associated with carbohydrate metabolism, as products of glycolysis such as acetyl-CoA, can be used in the synthesis of lipids. Between T1 and T2, several of the proteins that were differentially regulated play a known role in lipid metabolism; for example, KasA, KasB, and FabD levels increased between the time points, and MSMEG_2536 decreased (Supplementary Figure 6a). Furthermore, from T2 to T3, after treatment, proteins associated with lipid metabolism, such as Des, Glpk, MSMEG_5242, MSMEG_3580, and MSMEG_2597, showed increased abundance, whereas FadD9 (acyl-CoA synthetase) showed decreased abundance. These findings point to a tentative relationship between carbohydrate metabolism, lipid metabolism, and sublethal DETA-NO treatment.

Our analyses indicate that the levels of several proteins involved in signaling were affected between T1 and T2; specifically, the levels of PhoU, which is annotated as a virulence factor, were decreased, and the levels of the TetR transcription factor MSMEG_2553 were increased. The trend continued between T2 to T3, with a change in abundance of several virulence factors, such as RegX3, MtrA, DevR, MSMEG_3240, and MSMEG_5424, which are all members of two-component systems (see Fig. 4a). In addition to the increase of DevR abundance, other proteins controlled by the DosR regulon also increased in abundance, such as MSMEG_5243, MSMEG_5733, MSMEG_3945, MSMEG_5246, MSMEG_3940, MSMEG_5245, MSMEG_3942, MSMEG_3952, MSMEG_3950, and HspX (see Fig. 4c).

Among the members of the DosR response proteins, several were annotated as universal stress responders, including MSMEG_3945, MSMEG_3940, MSMEG_3950, MSMEG_5245, and MSMEG_5733. Notably, at T3, the abundance of proteins associated with DNA repair, such as Ku, MSMEG_2778, and MSMEG_5004, increased, suggesting DNA damage is induced by DETA-NO treatment. Evidence of protein expression perturbation from T2 to T3 was observed with the decrease in abundance of 11 ribosomal proteins. Additional accessory proteins involved in gene expression, such as Efp, MSMEG_1930, Rne, Rho, RecA, MSMEG_6892, and RpoC, were all found to decrease in abundance. These data are summarized in Table 2.

**Table 2:** Summary of a subset of differentially abundant proteins due to DETA-NO treatment, from all time points

Process	Uniprot accession	Gene name	Protein name	EC number	Fold change
Glycolysis/ gluconeogenesis	A0QU86	MSMEG_2116	PTS system, glucose-specific IIBC component (EC 2.7.1.-) (EC 2.7.1.69)	2.7.1.-; 2.7.1.69	0.22
Glycolysis/ gluconeogenesis	A0QU87	MSMEG_2117	Beta-glucoside-specific EII permease (PTS system sugar phosphotransferase component IIA) (EC 2.7.1.69)	2.7.1.69	0.22
Glycolysis/ gluconeogenesis	A0R170	orB	Alpha oxoglutarate ferredoxin oxidoreductase, beta subunit	N/A	0.27
Glycolysis/ gluconeogenesis	A0R171	MSMEG_4646	Pyruvate flavodoxin/ferredoxin oxidoreductase-like protein (EC 1.2.7.3) (pyruvate synthase)	1.2.7.3	0.31
Glycolysis/ gluconeogenesis	A0QUA6	pgm	Phosphoglucomutase PgmA (phosphoglucomutase, alpha-d-glucose phosphate-specific) (EC 5.4.2.2)	5.4.2.2	0.29
Glycolysis/ gluconeogenesis	A0QVJ6	MSMEG_2597	Aldehyde dehydrogenase (EC 1.2.1.3) (aldehyde dehydrogenase AldC) (EC 1.2.1.-)	1.2.1.3; 1.2.1.-	0.37
Glycolysis/ gluconeogenesis	A0QSN7	MSMEG_1543	Eptc-inducible aldehyde dehydrogenase (EC 1.2.1.3)	1.2.1.3	−0.36
Glycolysis/ gluconeogenesis	A0QNQ5	adhE1	Oxidoreductase, zinc-binding dehydrogenase family protein (zinc-type alcohol dehydrogenase (E subunit) AdhE) (EC 1.1.1.1)	1.1.1.1	0.30
Glycolysis/ gluconeogenesis	A0R2Z4	MSMEG_5287	Alcohol dehydrogenase (EC 1.1.1.1) (dehydrogenase)	1.1.1.1	0.35
Lipid metabolism	A0R0B4	kasA	3-oxoacyl-(Acyl-carrier-protein) synthase 1 KasA (3-oxoacyl-[acyl-carrier-protein] synthase 1) (EC 2.3.1.41)	2.3.1.41	0.37
Lipid metabolism	A0R0B5	kasB	3-oxoacyl-(Acyl-carrier-protein) synthase 1 KasA (3-oxoacyl-[acyl-carrier-protein] synthase 2) (EC 2.3.1.41)	2.3.1.41	0.28
Lipid metabolism	A0R0B2	fabD	Malonyl CoA-acyl-carrier protein transacylase (MCT) (EC 2.3.1.39)	2.3.1.39	0.57
Lipid metabolism	A0QVD5	MSMEG_2536	3-oxoacyl-[acyl-carrier-protein] reductase (EC 1.1.1.100) (short-chain dehydrogenase/reductase SDR)	1.1.1.100	−0.28
Lipid metabolism	A0R4B3	des	Acyl-acyl-carrier protein desaturase DesA1 (EC 1.14.19.2) (Fatty-acid desaturase)	1.14.19.2	0.68
Lipid metabolism	A0R726	glpK	Glycerol kinase (EC 2.7.1.30) (ATP:glycerol 3-phosphotransferase) (glycerokinase) (GK)	2.7.1.30	0.35
Lipid metabolism	A0R2V0	MSMEG_5242	Diacylglycerol o-acyltransferase (EC 2.3.1.20)	2.3.1.20	1.41
Lipid metabolism	A0QY95	MSMEG_3580	Antigen 85-C (EC 2.3.1.-)	2.3.1.-	0.48
Lipid metabolism	A0QVJ6	MSMEG_2597	Aldehyde dehydrogenase (EC 1.2.1.3) (Aldehyde dehydrogenase AldC) (EC 1.2.1.-)	1.2.1.3; 1.2.1.-	0.37
Lipid metabolism	A0QWI7	fadD9	Fatty-acid-CoA ligase FadD9 (EC 6.2.1.3) (NAD dependent epimerase/dehydratase family protein)	6.2.1.3	−0.49
Response regulators	A0R4B7	phoU	Phosphate-specific transport system accessory protein PhoU	N/A	−0.22
Response regulators	A0QVF2	MSMEG_2553	Transcriptional regulator, TetR family protein	N/A	0.25
Response regulators	Q9F868	regX3	Sensory transduction protein regX3	N/A	0.46
Response regulators	A0QTK2	mtrA	DNA-binding response regulator MtrA	N/A	0.34
Response regulators	A0R2V2	devR	LuxR family two-component response regulator (two-component transcriptional regulatory protein devr)	N/A	0.79
DosR regulon	A0QXB5	MSMEG_3240	DNA-binding response regulator, LuxR family protein	N/A	0.49
DosR regulon	A0R3C6	MSMEG_5424	Transcriptional regulator, TetR family protein	N/A	0.31
DosR regulon	A0R478	MSMEG_5733	Putative universal stress protein UspA (universal stress protein family protein)	N/A	0.58
DosR regulon	A0QZ91	MSMEG_3940	Universal stress protein family protein (UspA)	N/A	0.72
DosR regulon	A0QZ93	MSMEG_3942	Uncharacterized protein	N/A	0.86
DosR regulon	A0QZ96	MSMEG_3945	Universal stress protein family protein	N/A	0.61
DosR regulon	A0QZA2	MSMEG_3952	Uncharacterized protein	N/A	0.87
DosR regulon	A0QZA1	MSMEG_3950	Universal stress protein MSMEG_3950/MSMEI_3859 (USP MSMEG_3950)	N/A	0.90
DosR regulon	A0R2V1	MSMEG_5243	Helix-turn-helix motif (pyridoxamine 5’-phosphate oxidase-related, FMN-binding protein)	N/A	0.45
DosR regulon	A0R2V3	MSMEG_5245	Universal stress protein family protein (UspA)	N/A	0.72
DosR regulon	A0R2V4	MSMEG_5246	Uncharacterized protein	N/A	0.71
DosR regulon	A0QZ83	hspX	14 kDa antigen (Heat shock protein hspX)	N/A	1.05
DNA repair	A0R3S7	ku	Non-homologous end joining protein Ku	N/A	0.28
DNA repair	A0QW21	MSMEG_2778	Putative ribonuclease D (EC 3.1.-.-) (Ribonuclease D)	3.1.-.-	0.63
DNA repair	A0R267	MSMEG_5004	DNA repair exonuclease (DNA repair exonuclease SbcD)	N/A	0.43
Protein expression	A0QWR4	efp	Elongation factor P (EF-P)	N/A	−0.32
Protein expression	A0QTQ8	MSMEG_1930	DEAD/DEAH box helicase	N/A	−0.38
Protein expression	A0R218	rho	Transcription termination factor Rho (EC 3.6.4.-) (ATP-dependent helicase Rho)	3.6.4.-	−0.25
Protein expression	A0R152	rne	Ribonuclease E (RNase E) (EC 3.1.26.12)	3.1.26.12	−0.46
Protein expression	Q59560	recA	Protein RecA (recombinase A)	N/A	−0.33
Protein expression	A0R7F4	MSMEG_6892	Replicative DNA helicase (EC 3.6.4.12)	3.6.4.12	−0.58
Protein expression	A0QS66	rpoC	DNA-directed RNA polymerase subunit beta’ (RNAP subunit beta’) (EC 2.7.7.6) (RNA polymerase subunit beta’) (transcriptase subunit beta’)	2.7.7.6	−0.39
Protein expression	A0QSD1	rplC	50 S ribosomal protein L3	N/A	−0.25
Protein expression	A0QSG4	rplF	50 S ribosomal protein L6	N/A	−0.25
Protein expression	A0QS62	rplJ	50 S ribosomal protein L10	N/A	−0.25
Protein expression	A0QSG8	rplO	50 S ribosomal protein L15	N/A	−0.26
Protein expression	A0QSD6	rplV	50 S ribosomal protein L22	N/A	−0.25
Protein expression	A0QSD3	rplW	50 S ribosomal protein L23	N/A	−0.18
Protein expression	A0QV03	rpmB	50 S ribosomal protein L28	N/A	−0.31
Protein expression	A0QSL7	rpsD	30 S ribosomal protein S4	N/A	−0.25
Protein expression	A0QSG6	rpsE	30 S ribosomal protein S5	N/A	−0.27
Protein expression	A0QSD0	rpsJ	30 S ribosomal protein S10	N/A	−0.30

The process the protein is involved in as well as aliases is shown. The EC number, also displayed on the KEGG pathway diagrams, is shown along with the fold change of each protein

### Changes in *M. smegmatis* proteome induced by exposure to sublethal concentration of H_2_O_2_

After treatment with H_2_O_2_ between T1 and T2, an increase was observed in the abundance of proteins, such as Alpha oxoglutarate ferredoxin oxidoreductase and beta subunit orB, and a decreased level was observed of MSMEG_1543, a protein also involved in acetyl-CoA metabolism (Supplementary Figure 5A). Changes in the proteome during T1 to T2 suggest a potential deregulation of alanine, aspartate, and glutamate metabolism. From T2 to T3, an increase was observed in MSMEG_4646, a pyruvate synthase that like orB may suggest an increase in acetyl-CoA synthesis through the reaction 1.2.7.11 (Supplementary Figure 4). However, MSMEG_6297, which is also involved in acetyl-CoA metabolism, was observed to have a decreased abundance. The increased levels of proteins such as KasB and Des (Supplementary Figure 6B) suggest that exposure to H_2_O_2_ may also alter lipid metabolism and the cell envelope. H_2_O_2_ also appears to induce signaling responses because, from T1 to T2, a decrease in proteins such as the response regulator MSMEG_6236 (LuxR family transcriptional regulator) and an increased level of two TetR family transcription factors MSMEG_0532 and MSMEG_1611 was observed. From T2 to T3, evidence existed for further signaling perturbation, as indicated by the abundances of two-component system response regulators. Regulators such as RegX3, MtrA, and DevR, which are annotated as virulence factors, showed an increased abundance from T1 to T2, whereas other virulence factors such as LeuD and Mce4B decreased with treatment in the same time frame (Table 3).

**Table 3:** Summary of a subset of differentially abundant proteins due to Hydrogen Peroxide treatment, from all time points

Process	Uniprot accession	Gene name	Protein name	EC number	Fold change
Glycolysis/ gluconeogenesis	A0QSN7	MSMEG_1543	Eptc-inducible aldehyde dehydrogenase (EC 1.2.1.3)	1.2.1.3	−0.38
Glycolysis/ gluconeogenesis	A0R171	MSMEG_4646	Pyruvate flavodoxin/ferredoxin oxidoreductase-like protein (EC 1.2.7.3) (pyruvate synthase)	1.2.7.3	0.28
Glycolysis/ gluconeogenesis	A0R5S7	MSMEG_6297	Aldehyde dehydrogenase (EC 1.2.-.-) (aldehyde dehydrogenase)	1.2.-.-	−0.41
Glycolysis/ gluconeogenesis	A0R170	orB	Alpha oxoglutarate ferredoxin oxidoreductase, beta subunit	N/A	0,49
Lipid metabolism	A0R0B5	kasB	3-oxoacyl-(Acyl-carrier-protein) synthase 1 KasA (3-oxoacyl-[acyl-carrier-protein] synthase 2) (EC 2.3.1.41)	2.3.1.41	0.33
Lipid metabolism	A0R4B3	des	Acyl-acyl-carrier protein desaturase DesA1 (EC 1.14.19.2) (fatty-acid desaturase)	1.14.19.2	1.23
Response regulators	A0QPV5	MSMEG_0532	Transcriptional regulator, TetR family protein	N/A	0,27
Response regulators	A0QSV1	MSMEG_1611	Putative transcriptional regulatory protein (transcriptional regulator, TetR family protein, putative)	N/A	0.32
Response regulators	A0R5L8	MSMEG_6236	Response regulator, two-component system (two-component system, regulatory protein)	N/A	−0.51
Response regulators	A0R2V2	devR	LuxR family two-component response regulator (two-component transcriptional regulatory protein devr)	N/A	0.44
Response regulators	A0QUZ0	leuD	3-isopropylmalate dehydratase small subunit (EC 4.2.1.33) (alpha-IPM isomerase) (IPMI) (Isopropylmalate isomerase)	4.2.1.33	−0.42
Response regulators	A0R4N7	mce4B	MCE family protein MCE4b (virulence factor Mce family protein)	N/A	−0.23
Response regulators	A0QTK2	mtrA	DNA-binding response regulator MtrA	N/A	0.32
Response regulators	Q9F868	regX3	Sensory transduction protein regX3	N/A	0.30
DosR regulon	A0QZ91	MSMEG_3940	Universal stress protein family protein (UspA)	N/A	0.51
DosR regulon	A0QZ93	MSMEG_3942	Uncharacterized protein	N/A	0.54
DosR regulon	A0QZ96	MSMEG_3945	Universal stress protein family protein	N/A	0.43
DosR regulon	A0QZA1	MSMEG_3950	Universal stress protein MSMEG_3950/MSMEI_3859 (USP MSMEG_3950)	N/A	0.61
DosR regulon	A0QZA2	MSMEG_3952	Uncharacterized protein	N/A	0.63
DosR regulon	A0R2V1	MSMEG_5243	Helix-turn-helix motif (pyridoxamine 5’-phosphate oxidase-related, FMN-binding protein)	N/A	0.38
DosR regulon	A0R2V3	MSMEG_5245	Universal stress protein family protein (UspA)	N/A	0.43
DosR regulon	A0R2V4	MSMEG_5246	Uncharacterized protein	N/A	0.46
DosR regulon	A0R478	MSMEG_5733	Putative universal stress protein UspA (Universal stress protein family protein)	N/A	0.37
Redox homeostasis	A0R683	gltD	Glutamate synthase, NADH/NADPH, small subunit (EC 1.4.1.-) (Glutamate synthase, small subunit)	1.4.1.-	−0.24
Redox homeostasis	A0QWZ9	sufB	FeS assembly protein SufB	N/A	−0.62
Redox homeostasis	A0QTL3	MSMEG_1885	2Fe-2S iron-sulfur cluster binding domain protein (oxidoreductase FAD-binding domain protein)	N/A	0.91
Protein expression	A0QZ11	rbpA	RNA polymerase-binding protein RbpA	N/A	−0.26
Protein expression	A0QS66	rpoC	DNA-directed RNA polymerase subunit beta’ (RNAP subunit beta’) (EC 2.7.7.6) (RNA polymerase subunit beta’) (transcriptase subunit beta’)	2.7.7.6	−0.23
Protein expression	A0R218	rho	Transcription termination factor Rho (EC 3.6.4.-) (ATP-dependent helicase Rho)	3.6.4.-	−0.21
Protein expression	A0QVB8	rpsB	30 S ribosomal protein S2	N/A	−0.22
Protein expression	A0QSD7	rpsC	30 S ribosomal protein S3	N/A	−0.22
Protein expression	A0QSP9	rpsI	30 S ribosomal protein S9	N/A	−0.22
Protein expression	A0QSD0	rpsJ	30 S ribosomal protein S10	N/A	−0.21
Protein expression	A0QSL5	rpsM	30 S ribosomal protein S13	N/A	−0.25
Protein expression	A0QS62	rplJ	50 S ribosomal protein L10	N/A	−0.23
Protein expression	A0QSP8	rplM	50 S ribosomal protein L13	N/A	−0.23
Protein expression	A0QSG8	rplO	50 S ribosomal protein L15	N/A	−0.25
Protein expression	A0QSG5	rplR	50 S ribosomal protein L18	N/A	−0.28
Protein expression	A0QSG0	rplX	50 S ribosomal protein L24	N/A	−0.23

The processes the protein is involved in as well as aliases are shown. The EC number, also displayed on the KEGG pathway diagrams, is shown along with the fold change of each protein

**Fig. 5:**
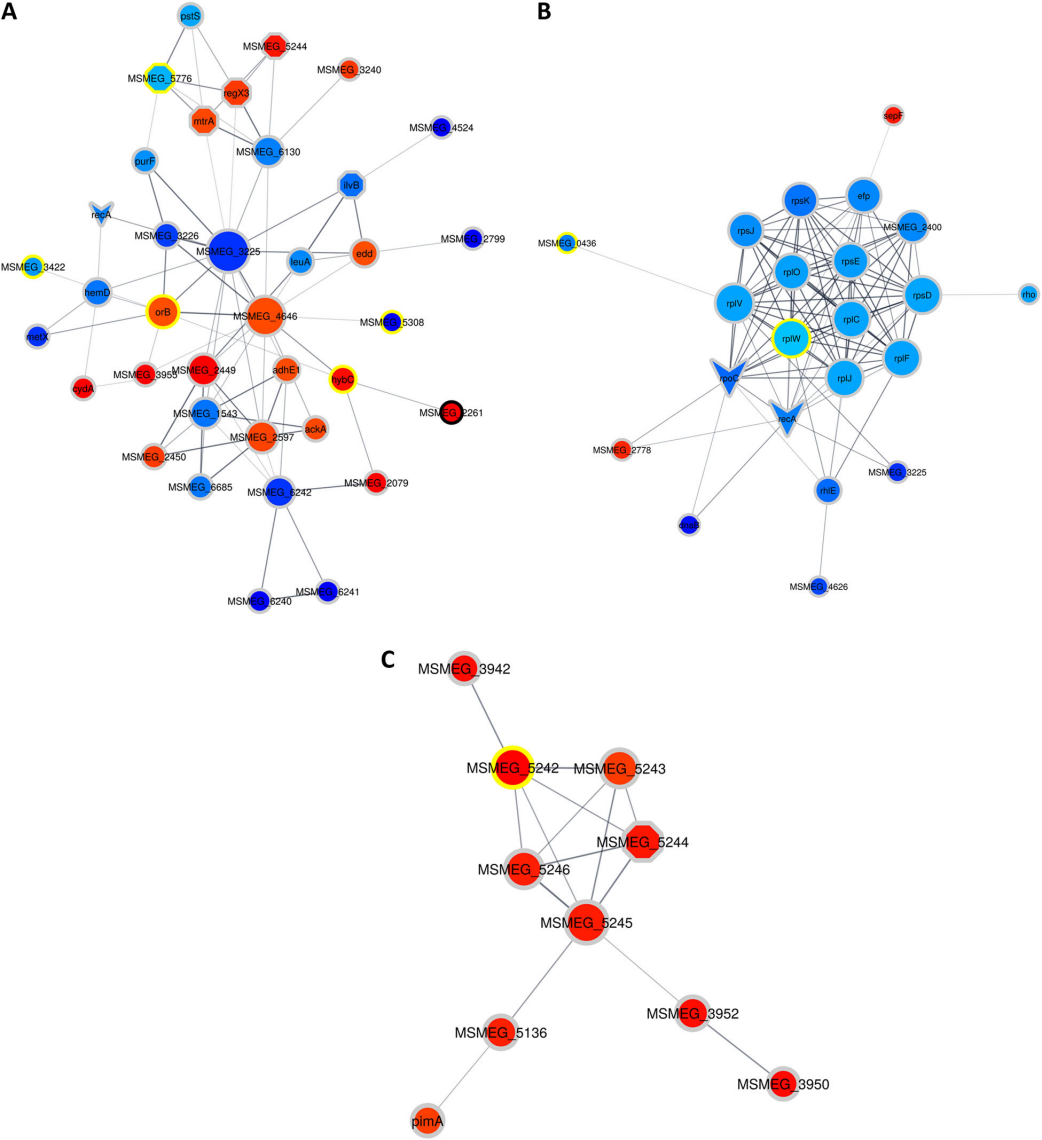
Clusters of associated proteins found to have significant differences in abundances as a result of H_2_O_2_ pre-exposure (as determined by String-db). Clusters were determined by the EAGLE algorithm using ClusterVis via Cytoscape. The shape of the node denotes if a protein is a known drug target or virulence factor. Octagons represent known virulence factors and arrowheads represent known drug targets, whereas ellipses represent proteins that are not known to be either. The color of the nodes denotes the protein expression relative to the previous time point on a gradient of dark blue to deep red, with dark blue indicating the relatively lowest expression and deep red indicating the relatively highest expression. The color of the ring surrounding the nodes denotes when the protein showed differential expression, with black rings indicating that in both comparisons (T1 and T3) the protein showed altered expression, the yellow rings indicate altered expression in only the first comparison (T1), and the gray rings indicate altered expression in only the second comparison (T3). **a** shows proteins associated with carbohydrate metabolism, involving pathways such as; glycolysis/gluconeogenesis and the citrate cycle. **b** shows ribosomal proteins and some proteins associated with gene expression, all of which are downregulated. **c** shows the DosR regulon related proteins, all of which are upregulated

Similar to the response with the DETA-NO treatment, between T2 and T3, DevR showed an increase with H_2_O_2_ treatment, which was accompanied by increased levels of other proteins controlled by the DevR regulon, including MSMEG_5243, MSMEG_5246, MSMEG_3952 (Putative NAD(P)H nitroreductase), MSMEG_5245 (Usp), MSMEG_3945 (Usp), MSMEG_3940 (UspA), MSMEG_3950 (Usp), and MSMEG_3942 (Fig. 5c and Table 3). Some differentially expressed members of the DosR regulon were annotated as universal stress responders such as MSMEG_3945, MSMEG_3940, MSMEG_3950, and MSMEG_5245. Another universal stress responder, MSMEG_5733, not annotated as part of the DevR regulon, also increased in abundance. Finally, the increased levels of proteins annotated with oxidoreductase activity were notable. These enzymes may play a role in restoring/maintaining the appropriate redox potential in the cell. Among this group, some proteins (MSMEG_1885, which shows increased abundance, SufB, and GltD, both show decreased abundances) were annotated as an iron-sulfur cluster binding protein. Proteins that modulate transcription and translation such as RpoC, RbpA, and Rho as well as 10 ribosomal proteins, observed as having decreased abundance between T2 and T3 (Fig. 5b and Table 3).

### Pre-exposure to DETA-NO or H_2_O_2_ enhanced survival during macrophage attack

**Fig. 6:**
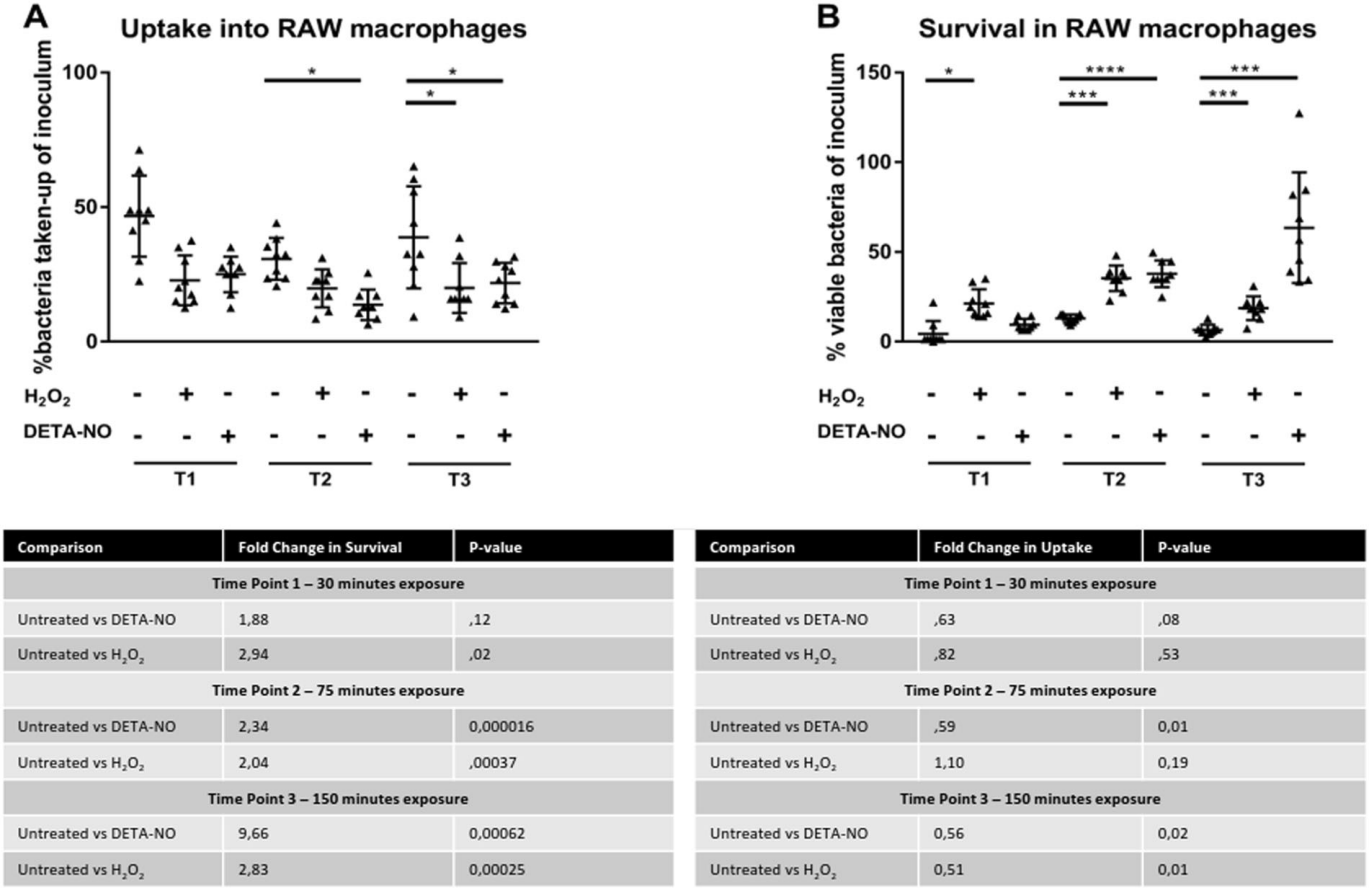
**a** CFU’s of uptake experiments. For T1, no significant differences between treatment conditions were observed. For T2, uptake following DETA-NO exposure was significantly lower than the untreated condition. At T3, both DETA-NO and H_2_O_2_ pre-exposure resulted in statistically significant lower uptake than the untreated condition. **b** CFU^’^s of survival experiments. For T1, only H_2_O_2_ pre-exposure resulted in a statistically significant increase in survival, with a 2.94-fold increase. For T2, pre-exposure with both DETA-NO and H_2_O_2_ resulted in a significant increase in survival, a 2.34- and 2.04-fold increase in survival, respectively. At T3 both DETA-NO and H_2_O_2_ pre-exposure resulted in a significant increase in survival compared with the untreated condition, with fold increases of 9.66 and 2.83, respectively. In both panels * represent statistically significant comparisons; * represents a *p* value < 0.05, *** represents a *p* value < 0.001 and **** represents a *p* value < 0.0001

To evaluate whether pre-exposure to sublethal concentrations of DETA-NO or H_2_O_2_ would confer a survival advantage during macrophage attack, we assessed macrophage uptake and bacterial survival at the three time points and compared the CFUs of pretreated bacteria and untreated bacteria. At T1, no significant difference occurred between treated and untreated bacteria for uptake into macrophages, as determined by a two-tailed student *t* test (see Fig. 6a). For survival at T1 in the DETA-NO treatment, no significant change was observed in survival despite a fold change increase of 1.88, whereas the H_2_O_2_ treatment showed a significantly higher (*p* = 0.02) rate of survival, with a fold increase of 2.94 (Fig. 6b). At T2, the bacteria pretreated with DETA-NO showed significantly decreased (*p* = 0.015) uptake into macrophages, with a 1.77-fold decrease. The H_2_O_2_ treatment showed no significant change in uptake (see Fig. 6a). Both DETA-NO and H_2_O_2_ pretreatments showed significant increases in survival with fold changes of 2.34 and 2.04, respectively (Fig. 6b). At T3, the bacteria treated with DETA-NO or H_2_O_2_ showed significantly decreased uptake of 1.78- and 1.94-fold decrease (Fig. 6a). At T3, DETA-NO pretreatment showed the greatest increase in survival, increasing 9.67-fold. H_2_O_2_ treatment resulted in a 2.83-fold increase in surviving bacteria to macrophage attack (Fig. 6b).

## Discussion

Previously, Li et al. demonstrated that 30 min of exposure to H_2_O_2_ was sufficient to induce widespread transcriptional adaptation in *M. smemgmatis*^14^. They also observed that 7 mM H_2_O_2_ was lethal after 3 h of exposure. These observations differ from those reported here. We observed 10 mM H_2_O_2_ as sublethal and able to induce a proteomic response as early as 30 min post exposure. These differences can be explained by the growth media supplements used in both cases. The supplement oleic acid albumin dextrose complex (OADC) used here contains catalase, which rapidly degrades H_2_O_2_. The supplement used by Li et al. was lacking catalase. The proteomic changes we observe here in cultures treated with H_2_O_2_ (Table 3) are the result of brief exposure to H_2_O_2_ and the likely signaling events associated with this brief exposure.

Redox stresses that mycobacteria encounter within hosts, such as ROS, RNS, hypoxia, and starvation, have been shown to trigger important adaptive changes in mycobacterial physiology that ultimately contribute to survival inside the host^3,25^. Understanding the mechanistic connection between the initial stimuli activated by signaling molecules such as H_2_O_2_ or NO and the subsequent events leading to bacterial cell responses requires a global view of the changes occurring at gene expression level^13,14^ as well as those happening at the protein level^16,26^. From this perspective, we aimed to establish a link between the changes induced in the *M. smegmatis* proteome and enhanced survival to macrophage attack. This was performed by exposing of *M. smegmatis* to sublethal concentrations of DETA-NO or H_2_O_2_ in liquid culture. Our results clearly indicate that the pretreatment of *M. smegmatis* to sublethal doses of DETA-NO or H_2_O_2_ conferred resistance to macrophage attack (Fig. 6b). Specifically, mycobacteria pretreated with H_2_O_2_ showed a higher survival rate at T2 and comparable survival rates between T1 and T3 (Fig. 6b). In contrast, pretreatment with DETA-NO resulted in an increase of bacterial survival throughout the time course assay, with the greatest improvement in survival occurring with bacteria having been exposed for an extended period to DETA-NO before infection (harvested at T3 for infection). Although H_2_O_2_ is often referred to as a main signaling molecule across species, in liquid culture, this compound degrades over time, either slowly by natural decomposition or more rapidly by the action of H_2_O_2_-scavenging enzymes. In addition, our complementary assays indicated that, under the studied conditions, 10 mM H_2_O_2_ persisted in the media at measurable levels for ~15 min (data not shown). This indicates that changes observed in the proteome throughout the time course are most likely owing to the earlier response signal pathways initiated during the first 30 min (T1). Previous work indicated that 40 min of exposure to sublethal concentrations of H_2_O_2_ (5 and 10 mM) had a strong effect on gene expression of *M. tuberculosis*^13^. By comparison, in *M. smegmatis* exposure to 0.2 mM H_2_O_2_ changed the expression of ~10% of the genome, whereas 29% of the genes were significantly changed in response to 7 mM H_2_O_2_^14^. Here, our proteomic data revealed that important transcriptional regulatory proteins showed a change in abundance after exposure to H_2_O_2_. For instance, two proteins annotated as TetR transcriptional regulators increased from T1 to T2 after H_2_O_2_ exposure only. TetR family like proteins control genes whose products are associated with several cellular functions including stress response, multidrug resistance, metabolic modulation, efflux pumps, and pathogenesis^27^. In addition, the increased abundance of two-component system (TCS) regulatory protein MSMEG_6236 indicates that the initial presence of H_2_O_2_ altered the phosphorelay signal transduction pathways also likely to impact gene expression. We therefore hypothesize that during T1, the H_2_O_2_ triggered cellular responses that promote differential gene expression, preparing *M. smegmatis* for an eventual oxidative stress. In culture, the responses initiated at T1 most likely resulted in some of those protein changes observed later at T2 and T3, including the DevR regulon. Possibly, specific signal pathways initiated by H_2_O_2_ in vitro are then further propagated once the bacterium is in contact with the macrophage. This would, in part, account for higher survival of T1-treated *M. smegmatis* in comparison with the untreated control. Interestingly, when compared with the proteome of *M. smegmatis* treated with sublethal doses of rifampicin^21^, 30 of 34 common differentially abundant proteins were found to have opposite changes in abundance. Phenotypically, *M. smegmatis* pretreated with rifampicin resulted in lower macrophage survival rates when compared with the control (data not shown). This further supports the potential role of the above-mentioned proteins in macrophage survival.

### Sensors and response regulators of ROS and NO

During T2 and T3, evidence was observed of the regulation of redox sensors that are known to drive important protective response in mycobacteria. Herein, our results strongly indicate that the DevR regulon responds to both NO or H_2_O_2_ insult at the protein level. In *M. tuberculosis*, the DevR regulon comprises 48 genes whose expression is altered under hypoxic conditions and exposure to NO^28,29^. In *M. smegmatis*, DevR has a key role in adaptation to the oxygen-starved stationary phase and resistance to environmental stresses via the induction of three ubiquitin proteasome system proteins, nitroreductase, and HspX^30^. By comparison, here we observed the induction of 11 DevR regulated proteins in response to H_2_O_2_ or DETA-NO, which is consistent with previous observations^17^. This subgroup of proteins included five USPs, one LuxR TC response regulator, nitroreductase like proteins MSMEG_3952, MSMEG_5243, one pyridoxamine 5-phosphate oxidase-related PdxH, and hypoxia-induced HspX. As mentioned earlier, treatment with H_2_O_2_ slowed down the growth rate, whereas DETA-NO had no effect on culture growth, so in *M. smegmatis* the induction of DevR could be part of an early defense mechanism to redox or NO stimuli, whereas mycobacteria are not necessary committed to a dormant state. In this regard, the induction of DevR-regulated defense proteins, such as Rv2623, PdxH, and HspX, certainly contributed to the survival of the pathogen in macrophages^31,32^.

Notably, evidence exists of transcriptional activity of the DevR regulon together with increased levels of other important mycobacterial responses regulators (RR) such as RegX3 and MtrA. In mycobacteria, the response to environmental stimulus is in part mediated by TCSs. These sensor systems consist of a paired histidine kinase coupled to a respective response regulator RR. The sensing of a signal by the kinase triggers autophosphorylation on a histidine residue, which then transfers the phosphate to an aspartate residue of the cognate RR, facilitating binding to its specific DNA sequence resulting in subsequent transcriptomic changes. SenX3–RegX3 is expressed during phosphate starvation, whereas MtrAB is the only essential TCS known so far. Recent evidence showed that RegX3 and MtrA belong to the OmpR family that shares a conserved DNA-binding motif. Of interest, in *M. tuberculosis*, the survival of the DNA-binding mutant RegX3 strain is compromised in macrophages^33^. Preliminary work in our laboratory indicates that *M. smegmatis* treated with sublethal doses of rifampicin resulted in a decrease in the abundance of both RegX3 and MtrA^21^, as well as lower rates of survival in macrophages (data not shown). Overall, the information gathered here suggest that sublethal concentrations of NO or H_2_O_2_ initiate a concerted network of phosphorylation events that ultimately modulate gene expression responses. During the adaptation of *M. tuberculosis* to the macrophage microenvironment, the DosR regulon is upregulated along with genes associated with fatty-acid metabolism, and in addition, ribosomal genes are downregulated^34^. These findings are similar to our own observations and suggest that a sublethal oxidative or nitrosative stress simulates in part the macrophage microenvironment. Although further research is needed for confirmation, possibly the modulated gene expression may offer an initial advantage to the bacteria during macrophage attack.

At T3, protein changes overlap between the two treatments were greater than those unique to either treatment, although the survival rates were considerably higher for bacteria cells pretreated with DETA-NO. This finding not only reinforces the concept that these two compounds initiate different molecular responses in the mycobacteria but also suggest that, in this case, specific responses to DETA-NO or H_2_O_2_ are the basis of the differences seen between survival rates of the treated versus the untreated bacteria once inside the macrophage. The bacteria treated with DETA-NO for 150 min (harvested at T3) before infecting the macrophages were found to have the highest survival rate.

### DETA-NO pretreatment results in altered expression of proteins involved in lipid metabolism and, potentially, in altered lipid metabolism

A particularly striking observation is that the abundance of several proteins involved in lipid metabolism was altered exclusively at T3 in response to the DETA-NO treatment; these include FabD, KasA, PimA, MSMEG_3580, and MmsA. Both FabD and KasA are involved in the early steps for mycolic acid synthesis^35^. In *M. tuberculosis*, the FabD transfers a malonate moiety to an acyl-carrier protein before it enters into the fatty-acid synthase—II (FAS-II) pathway for merochain biosynthesis^36^. KasA is a member of the FAS-II biosynthetic pathway, where it is responsible for lengthening the merochain of mycolic acids^35,36^. In addition, it has been shown that *Rhodococcus equi* KasA mutants have shorter mycolic acid chain lengths, and these mutants were attenuated in both macrophage and mouse infection models^37^. PimA, an enzyme that transfers a mannosyl residue from GDP-d-mannose across the plasma membrane to phospho-*myo-*inositol on the inner side of the plasma membrane, has been shown to be essential for growth of *M. tuberculosis* both in vitro and in vivo^38,39^. In addition, depletion of PimA by gene silencing allowed for infections in mouse lungs to be effectively cleared^38^. MmsA was shown to be upregulated in a long-term macrophage infection model, coinciding with the increase of *M. tuberculosis* growth, suggesting that MmsA plays a role in the adaptation to the phagosomal environment^34^. These results tentatively suggest an important role for lipid metabolism during a mycobacterial infection, possibly mediated through the alteration of membrane lipids such as mycolic acids and phosphatidyl-*myo-*inositol, which can serve to protect the infecting bacterium from macrophage attack. The increased survival rate of DETA-NO pre-exposed bacteria (T3, see Fig. 6b) could be owing to the increased abundance of various proteins involved in lipid synthesis, causing structural changes at the microbial lipid layer and improving survival rates within the macrophage.

## Conclusion

Findings presented in this work show a large overlap in the proteome changes in *M. smegmatis* when exposed to DETA-NO and H_2_O_2_, as suggested by transcriptomic data. In both treatment conditions, the abundance of proteins involved in lipid metabolism, as well as the DosR response, were affected, and the survival to subsequent macrophage attack was increased as a result of the phenotypic proteome adaption that is characterized here. DETA-NO treatment resulted in the increased abundance of more proteins related to lipid metabolism when compared to H_2_O_2_ treatment, and the survival rates for the DETA-NO pre-exposed bacteria was higher. These results point to the potential importance of changes in the mycobacterial lipidome that are driven by phenotypic adaption of the proteome in the context of infections. In conjunction with preliminary work in our laboratory, these findings indicate that sublethal concentrations of bactericidal stressors cause different signaling pathways to be activated, leading to varying phenotypic responses.

## Materials and methods

### Bacterial cell culture

*M. smegmatis mc (2)155* was grown in shaking liquid batch culture at 37 °C in Middlebrook 7H9 (Beckton Dickinson (BD)) supplemented with 10% (v/v) OADC (BD), 0.5% (v/v) glycerol, and 0.05% (v/v) Tween-20. Liquid cultures were grown from single colonies, cultured on 7H10 (BD) agar plates, supplemented with 0.5% (v/v) glycerol and 10% (v/v) OADC (BD).

### Growth curves

Mid-log phase *M. smegmatis* cultures (OD_600_ ~ 1.2) were exposed to a single dose of H_2_O_2_ (Sigma Aldrich, St. Louis, USA) or the DETA/NO (Sigma Aldrich, St. Louis, USA). Doses of 0, 10, 50, 100, and 200 mM H_2_O_2_ and 0.05 mM DETA/NO were used. The OD_600_ was monitored after exposure of the cultures to respective H_2_O_2_/DETA concentrations at 15-minute intervals for 2 hours, 30-minute intervals for an additional 2 h, and a final reading 5 h after oxidant exposure.

### Protein extraction

*M. smegmatis* cultures were grown to the mid-log phase in the conditions described above, in biological triplicate for each condition, and then treated with either H_2_O_2_ or DETA-NO at a concentration of 10 mM and 0.05 mM, respectively. Bacterial pellets were snap frozen in liquid nitrogen and stored at − 80 °C, until lysis. Frozen bacterial pellets were thawed on ice, in lysis buffer (1% m/v sodium dodecyl sulfate, 1.5% m/v sodium deoxycholate, 1× protease inhibitor, and 7.5 µl lysozyme in 0.5 m Tris-HCL at pH 7). The resulting lysate was centrifuged at 13,000 rpm for 5 min to sediment the cell debris. The supernatant was passed through a 20 µm filter. Protein was precipitated from the supernatant by chloroform/methanol precipitation, and the protein pellet was resuspended in denaturation buffer (6 M urea, 2 M thiourea in 10 mM Tris at pH 8). Protein was quantified using the modified Bradford assay^40^. Proteins were digested with trypsin (Promega) at a ratio of trypsin to protein of 1:50 w/w at room temperature for 16 h. Peptides were desalted using C18 solid phase extraction.

### Liquid chromatography with tandem mass spectrometry (LC/MS/MS analysis)

For label-free quantification, samples were separately injected into a Dionex UltiMate 3500 RSLCnano system (Thermo Scientific, Waltham, Massachusetts) coupled to an Orbitrap Q Exactive mass spectrometer (Thermo Scientific) for analysis. Each sample was loaded onto a 2 cm trap (packed in-house, 5 μm beads, 100 Å pores, Luna beads by Phenomenex) at a flow rate of 300 nL/min and subsequently run on a 20 cm C18-reversed phase analytical column (packed in-house, 5 μm beads, 100 Å pores) at 40 °C. Approximately 600 ng of peptides was loaded in each LC/MS run. Elution from the column occurred over a 190 min segmented gradient consisting of an increasing ratio of 2% acetonitrile acidified with 0.1% formic acid (FA) (buffer B), to H_2_O acidified with 0.1% FA (buffer A): 1% B from 0 to 10 min, increasing up to 6% at 12 min, to 35% at 130 min, and to 80% at 135 min and continuing at 80% until 150 min, before decreasing from 80 to 2% at 152 min. Each run had a built-in wash at the end, increasing to 50% B at 167 min and continuing at this concentration until 169 min, at which time the gradient decreased to 2% for the final 20 min. Before entering the mass analyzer, the eluents were subjected to electrospray ionization. Mass spectra were acquired in a data-dependent manner, with automatic switching between MS and MS/MS scans using a top-10 method. MS spectra were acquired at a resolution of 70,000 with a target value of 3 × 10^6^ or a maximum integration time of 250 milliseconds (ms). The scan range at the MS level was limited to 300–1750 *m/z* and high-energy collision dissociation used for peptide fragmentation, with the energy set at normalized collision energy 28. Multiple charge exclusion was used (unassigned, 1, 5–8, > 8). MS/MS spectra were acquired at a resolution of 17,500, with a target value of 5 × 10^6^ or a maximum integration time of 80 ms. The scan range at the MS/MS level was limited from 200 to 2000 *m/z*. The fixed first *m/z* was 200 to 2000 *m/z*, and the isolation window was 2 *m/z*.

### Peptide identification and protein inference

Raw spectral data were processed using MaxQuant^41,42^ software package (version 1.5.7.4) for protein and peptide identification and quantitation with the below-described parameters. Trypsin/P was selected as the protease with a maximum of two missed cleavages allowed. Quantitation by MaxLFQ algorithm was selected with a minimum ratio count of two peptides. Protein ratios were calculated from unique and razor peptides. Protein N-terminal acetylation and methionine oxidation were set as variable modifications, and cysteine carbamidomethylation selected as a fixed modification. The Andromeda search engine was used for identification of the proteins and protein groups from the UniProt *M*. *smegmatis* reference proteome (FASTA, downloaded 02/05/2017). Mass tolerance for the precursor ions in the initial search was set at 20 ppm, and in the main search, at 4.5 ppm tolerance. The default protein-level false discovery rate setting of 1% was retained. Only proteins with a minimum of one unique peptide were accepted. The minimum length of acceptable identified peptides was set to seven amino acids. The option to match retention times between runs was selected to maximize identifications. MS/MS spectra were matched against a decoy database and reverse hits (i.e., spectral mapping to the decoy databases) and probable contaminants filtered from the protein list prior to statistical analysis.

### Experimental design and statistical rationale

In this study, 27 samples were analyzed, corresponding to three biological replicates of nine conditions. In brief, the nine conditions were divided into three time points and three treatment conditions: 10 mM H_2_O_2_, 0.05 mM DETA-NO, and untreated (control). For a graphical representation of the experimental workflow see Supplementary Figure 1.

An expression fold change cutoff was employed, so protein groups that changed by less than one standard deviation around the median of the fold change between conditions were excluded from the *t* test. These values can be seen in Table 1. Protein groups with a *p* value < 0.05 were considered to have a statistically significant change in abundance. Samples within each treatment condition were compared between time points to assess the response over time to treatments with the oxidative stressors. Changes found between corresponding time points within the control were subtracted from the treatment conditions, as those were likely to be related to normal growth. Remaining changes were assumed to be specific to the oxidative stressor condition.

Proteins with altered expression between the first and second time points, as well as between the second and third time points, were analyzed simultaneously while the time dependence was still preserved. Proteome changes owing to treatment were analyzed using String-db^43^ protein association networks generated in Cytoscape^44^ to visualize the data. The networks were clustered using the EAGLE algorithm via ClusterViz^45^ and were used to query String-db for a general overview of the proteomic changes. To gain further insight into the dysregulation at the metabolic level, we used the KEGG^46^ pathway mapper to visualize and analyze the metabolic changes associated with treatment.

Patients suffering from acute pulmonary tuberculosis expel droplets containing mycobacteria, which are unlikely to be naive to sublethal oxidative and or nitrosative stress^3,47^. These non-naive bacteria can then be inhaled by others and establish a new infection, or dormant bacilli may undergo reactivation to establish an active infection. In both cases understanding the adaptation to sublethal stresses are important. Our study aims to use *M. smegmatis* as a model organism to study how prior adaptation to sublethal oxidative or nitrosative stress influences bacterial survival within macrophages.

### Murine macrophage culture

Raw 264.7 cells were cultured in high-glucose Dulbecco’s Modified Eagle’s Medium (DMEM, Sigma), supplemented with 10% heat inactivated fetal bovine serum (FBS) at 37 ^o^C in an atmosphere of 5% CO_2_ and 95% O_2_. All cells used were between passage 21 and 25. Infection of Raw 264.7 macrophages with H_2_O_2_ or DETA-NO pretreated *M. smegmatis*

For infection assays 25000 cells were seeded onto 24-well plates and incubated for 72 h pre-infection in DMEM, 10% FBS. Murine Interferon-γ (mIFN-γ, 250 U/ml) was added 48 h after seeding. After 72 h cells were washed with pre-warmed phosphate-buffered saline (PBS) and incubated with DMEM, 10% FBS, 250 U/ml mIFN-γ, and *M. smegmatis* at a multiplicity of infection of 4:1, for 3 hours. The cells were washed five times with pre-warmed PBS, to remove extracellular bacteria, and incubated in DMEM, 10% FBS and 250 U/ml mIFN-γ. After a further 21 h cells were washed five times with pre-warmed PBS, to once again remove extracellular bacteria, before cell lysis for colony forming unit (CFU) determination. Before infection *M. smegmatis* cultures were treated with 10 mM H_2_O_2_ or 0.05 mM DETA-NO, or left untreated as described above. At 30, 75, or 150 min post exposure to the respective treatment condition, *M. smegmatis* cultures were harvested for infection.

### Macrophage infections

After infection, CFUs were used to assess mycobacterial survival. Raw 264.7 cells were washed after incubation with *M. smegmatis* for either three hours (uptake) or 24 h (survival) as described above. The cells were lysed by incubation in PBS with 0.1% triton X-100 for ten min on a rotary shaker at 200 rpm. The lysate was diluted and cultured for 48 h on 7H10 agar (0.5% glycerol and 10% OADC) at 37 °C in a standing incubator. The inoculum was cultured and used to normalize uptake values. Three independent experiments were performed, each experiments was performed in technical triplicate. CFU counts for uptake were used to normalize CFU counts for survival, according to the equations below:

1 $$Normalised\,uptake = \frac{{CFU_U \times DF_U}}{{CFU_I \times DF_I}}$$

2 $$Normalised\,survival = \frac{{\begin{array}{*{20}{c}} {CFU_S \times DF_S} \end{array}}}{{CFU_U \times DF_U}}$$

CFU_U_ = colony-forming units from uptake experiments, 3 hours post infection

DF_U_ = dilution factor used for plating uptake experiments, 5000

CFU_I_ = colony-forming units for the inoculum used for infections

DF_I_ *=* dilution factor used for plating the inoculum, 20000

CFU_S_ = colony-forming units for the survival experiments, 24 h post infection

DF_s_ = dilution factor used for plating survival experiments, 80000

## Supplementary information

Parameters used for the MaxQuant search and subsequent protein quantitation

Supplementary Figures

## Data Availability

The mass spectrometry proteomics data have been deposited to the ProteomeXchange Consortium via the PRIDE^48^ partner repository with the dataset identifier PXD010020.

## References

[1] Bhat SA (2012). The mechanism of redox sensing in Mycobacterium tuberculosis. Free Radic. Biol. Med..

[2] Lee PPW (2008). Susceptibility to mycobacterial infections in children with X-linked chronic granulomatous disease. Pediatr. Infect. Dis. J..

[3] Gengenbacher M, Kaufmann SHE (2012). Mycobacterium tuberculosis: success through dormancy. FEMS Microbiol. Rev..

[4] Kumar A (2011). Redox homeostasis in mycobacteria: the key to tuberculosis control?. Expert. Rev. Mol. Med..

[5] Landes MB, Rajaram MVS, Nguyen H, Schlesinger LS (2015). Role for NOD2 in Mycobacterium tuberculosis-induced iNOS expression and NO production in human macrophages. J. Leukoc. Biol..

[6] D’Autréaux B, Toledano MB (2007). ROS as signalling molecules: mechanisms that generate specificity in ROS homeostasis. Nat. Rev. Mol. Cell Biol..

[7] Bhaskar, A. et al. Reengineering redox sensitive GFP to measure mycothiol redox potential of Mycobacterium tuberculosis during Infection. *PLoS. Pathog*. **10**, (2014). 10.1371/journal.ppat.1003902 PMC3907381 24497832

[8] Brugarolas P (2012). The oxidation-sensing regulator (MosR) is a new redoxdependent transcription factor in Mycobacterium tuberculosis. J. Biol. Chem..

[9] Singh A (2007). Mycobacterium tuberculosis WhiB3 responds to O2 and nitric oxide via its [4Fe-4S] cluster and is essential for nutrient starvation survival. Proc. Natl. Acad. Sci..

[10] Singh, A. et al. Mycobacterium tuberculosis WhiB3 Maintains redox homeostasis by regulating virulence lipid anabolism to modulate macrophage response. *PLoS. Pathog*. **5**, (2009). 10.1371/journal.ppat.1000545 PMC2718811 19680450

[11] den Hengst CD, Buttner MJ (1780). Redox control in actinobacteria. Biochim. Biophys. Acta.

[12] Garbe TR, Hibler NS, Deretic V (1996). Response of Mycobacterium tuberculosis to reactive oxygen and nitrogen intermediates. Mol. Med..

[13] Voskuil MI, Bartek IL, Visconti K, Schoolnik GK (2011). The response of Mycobacterium tuberculosis to reactive oxygen and nitrogen species. Front. Microbiol..

[14] Li X, Wu J, Han J, Hu Y, Mi K (2015). Distinct responses of mycobacterium smegmatis to exposure to low and high levels of hydrogen peroxide. PLoS ONE.

[15] Schubert, O. T. & Aebersold, R. in *Prokaryotic Systems Biology* (eds. Krogan PhD, N. J. & Babu PhD, M.) 235–254 (Springer International Publishing, 2015).

[16] Schubert OT (2015). Absolute proteome composition and dynamics during dormancy and resuscitation of mycobacterium tuberculosis. Cell. Host. Microbe.

[17] Cortes, T. et al. Delayed effects of transcriptional responses in Mycobacterium tuberculosis exposed to nitric oxide suggest other mechanisms involved in survival. *Sci. Rep*. **7** 1–9 (2017). 10.1038/s41598-017-08306-1 PMC5557973 28811595

[18] Tyagi JS, Sharma D (2002). Mycobacterium smegmatis and tuberculosis. Trends Microbiol..

[19] Shiloh MU, Champion PA (2010). NIH Public Access.. J. Health Commun..

[20] Reyrat J, Kahn D (2001). Mycobacterium smegmatis: an absurd model for tuberculosis?. Trends Microbiol..

[21] Giddey, A. D. et al. A temporal proteome dynamics study reveals the molecular basis of induced phenotypic resistance in Mycobacterium smegmatis at sub-lethal rifampicin concentrations. *Sci. Rep.* **7**, 43858 (2017). 10.1038/srep43858 PMC5338346 28262820

[22] Albeldas, C. et al. Global proteome and phosphoproteome dynamics indicate novel mechanisms of vitamin C induced dormancy in Mycobacterium smegmatis. *J. Proteomics*. **180**, 1–10 (2017). 10.1016/j.jprot.2017.10.006 29038038

[23] He W, Frost MC (2016). Direct measurement of actual levels of nitric oxide (NO) in cell culture conditions using soluble NO donors. Redox Biol..

[24] Tyanova, S. et al. The Perseus computational platform for comprehensive analysis of (prote)omics data. *Nat. Meth.* **13**, 731–40(2016). 10.1038/nmeth.3901 27348712

[25] Lipworth, S. et al. Defining dormancy in mycobacterial disease. **99**, 131–42 (2016). 10.1016/j.tube.2016.05.006 27450015

[26] Schubert OT (2014). NIH Public Access..

[27] Ramos JL (2005). The TetR family of transcriptional repressors. Microbiol. Mol. Biol. Rev..

[28] Saini, D. K., Malhotra, V. & Tyagi, J. S. Cross talk between DevS sensor kinase homologue, Rvc, and DevR response regulator of Mycobacterium tuberculosis. **565**, 75–80 (2004). 10.1016/j.febslet.2004.02.092 15135056

[29] Saini DK (2004). DevR-DevS is a bona fide two-component system of Mycobacterium tuberculosis that is hypoxia-responsive in the absence of the DNA-binding domain of DevR. Microbiology.

[30] Toole RO (2003). A two-component regulator of universal stress protein expression and adaptation to oxygen starvation in Mycobacterium smegmatis. Society.

[31] Ankisettypalli K, Cheng JJY, Baker EN, Bashiri G (2016). PdxH proteins of mycobacteria are typical members of the classical pyridoxine/pyridoxamine 5′-phosphate oxidase family. FEBS Lett..

[32] Monahan IM, Betts J, Banerjee DK, Butcher PD (2001). Differential expression of mycobacterial proteins following phagocytosis by macrophages. Microbiology.

[33] Banerjee SK (2016). Targeting multiple response regulators of Mycobacterium tuberculosis augments the host immune response to infection. Sci. Rep..

[34] Russell, D. G., Rohde, K. H., Veiga, D. F. T. & Caldwell, S. Linking the transcriptional profiles and the physiological states of Mycobacterium tuberculosis during an extended intracellular infection. Plos Pathog **8**, e1002769 (2012). 10.1371/journal.ppat.1002769 PMC3380936 22737072

[35] Nataraj V (2015). Mycolic acids: deciphering and targeting the Achilles' heel of the tubercle bacillus. Mol. Microbiol..

[36] Takayama, K., Wang, C. & Besra, G. S. Pathway to synthesis and processing of mycolic acids in mycobacterium tuberculosis. Genetic analysis of synthesis and processing of mycolic acid. **18**, 81–101 (2005). 10.1128/CMR.18.1.81-101.2005 PMC544180 15653820

[37] Sydor T (2013). Diversion of phagosome trafficking by pathogenic Rhodococcus equi depends on mycolic acid chain length. Cell Microbiol..

[38] Boldrin F (2014). The phosphatidyl-myo-inositol mannosyltransferase PimA is essential for Mycobacterium tuberculosis growth in vitro and in vivo. J. Bacteriol..

[39] Puzo, G., Brennan, P. J., Gicquel, B. & Jackson, M. Definition of the first mannosylation step in phosphatidylinositol mannoside synthesis. PimA is essential for growth of mycobacteria. *J. Biol. Chem*. **277**, 31335–31344 (2002). 10.1074/jbc.M204060200 12068013

[40] Ramagli LS (1999). Quantifying protein in 2-D PAGE solubilization buffers. Methods Mol. Biol..

[41] Cox J, Mann M (2008). MaxQuant enables high peptide identification rates, individualized p.p.b.-range mass accuracies and proteome-wide protein quantification. Nat. Biotech..

[42] Tyanova S, Temu T, Cox J (2016). The MaxQuant computational platform for mass spectrometry-based shotgun proteomics. Nat. Protoc..

[43] Szklarczyk D (2015). STRINGv10: protein–protein interaction networks, integrated over the tree of life. Nucleic Acids Res..

[44] Cline MS (2007). Integration of biological networks and gene expression data using Cytoscape. Nat. Protoc..

[45] Wang J, Chen G, Li M, Wu F, Pan Y (2014). ClusterViz: a Cytoscape APP for luster analysis of biological. Network.

[46] Kanehisa, M., Sato, Y., Kawashima, M., Furumichi, M. & Tanabe, M. KEGG as a reference resource for gene and protein annotation. *Nucleic Acids. Res.* **44**, 457–462 (2016). 10.1093/nar/gkv1070 PMC4702792 26476454

[47] Kaufmann SHE (2001). How can immunology contribute to the control of tuberculosis?. Nat. Rev. Immunol..

[48] Vizcaíno JA (2016). 2016 update of the PRIDE database and its related tools. Nucleic Acids Res..

