# Supplementary material for: Associating H2O2-and NO-related changes in the proteome of Mycobacterium smegmatis with enhanced survival in macrophage
Source: Emerg Microbes Infect. 2018 Dec 13;7:212. doi: 10.1038/s41426-018-0210-2 (PMC6292918; doi:10.1038/s41426-018-0210-2)
Supplement: Supplementary file 2 — Supplementary Figures [file 41426_2018_210_MOESM2_ESM.pdf]

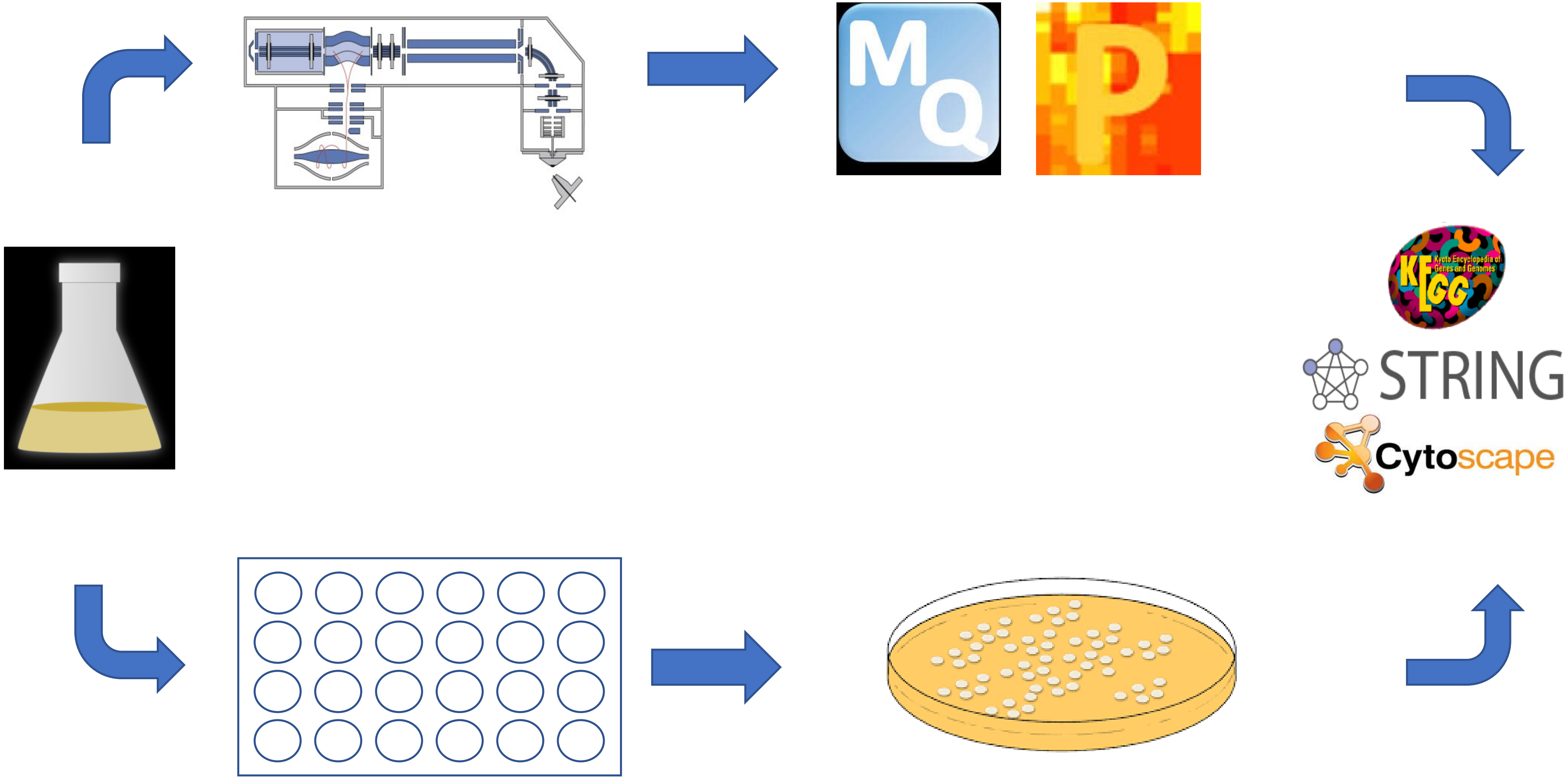

Figure S - 1. Briefly, M. smegmatis cultures were grown in 7H9 media at 37C, before being treated with either H2O2 or DETA-NO or left Untreated. At three time-points subsequent to treatment cultures were harvested for proteomic measurement. MaxQuant was used to identify and quantify protein groups, the Perseus software environment was used to perform quality control and the statistical analysis of the MaxQuant output. Additionally, M. smegmatis cultures treated identically to those used for proteomic measurement, were used to infect Raw 246.7 cells. Subsequent to infection, surviving M. smegmatis cells were quantified by counting colony forming units. The information gained from the infection assays were used to give context to the protein groups identified as statistically significant, using various platforms including KEGG, Cytoscape and STRINGdb.

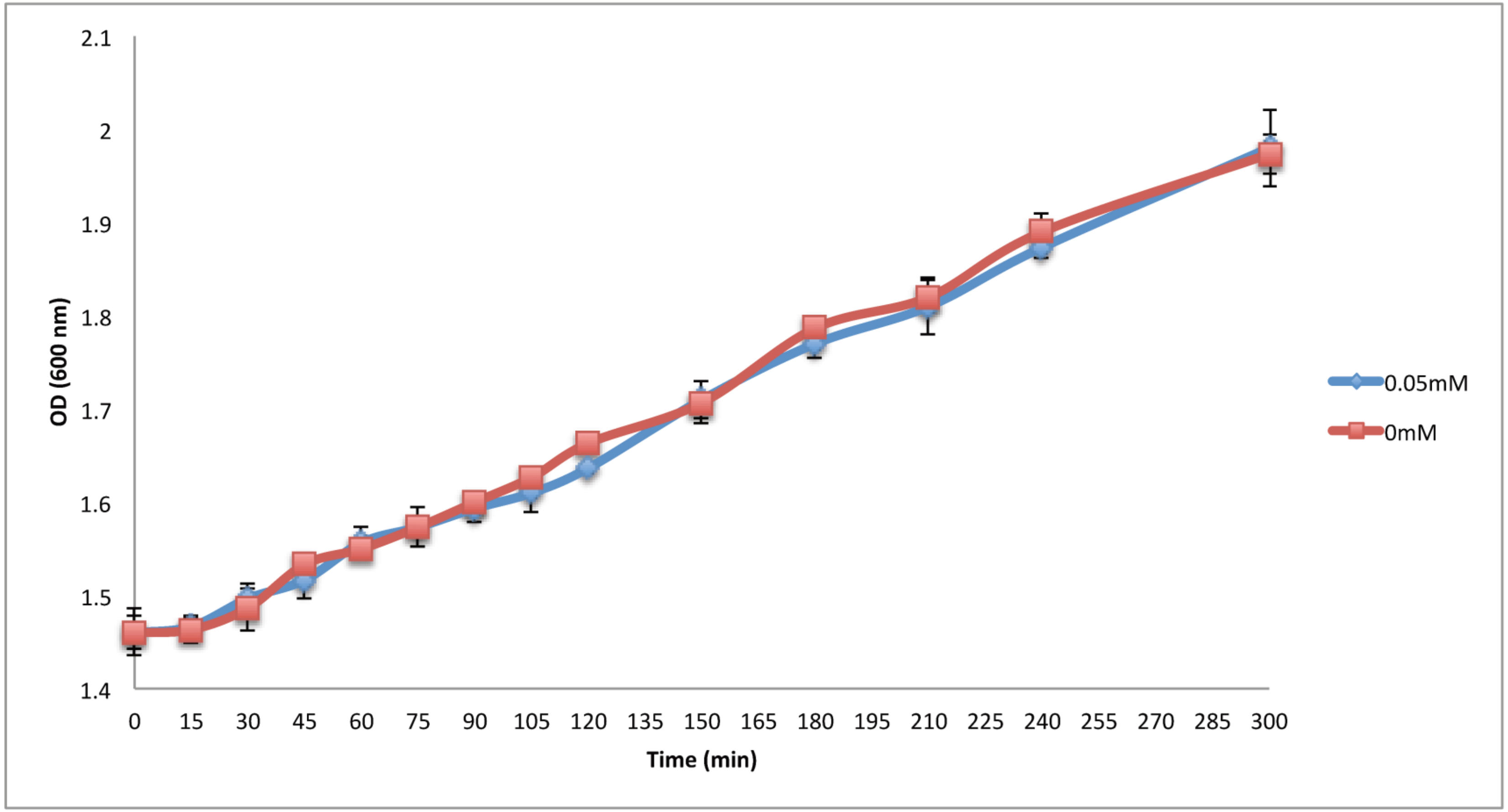

Figure S - 2, shows the growth curves of M. smegmatis cultures growth with (blue) or without (red) DETA-NO. At the concentration (0,05mM) previously reported by Voskuil, et al as sub-lethal in M. tuberculosis, shows no effect on growth on M. smegmatis.

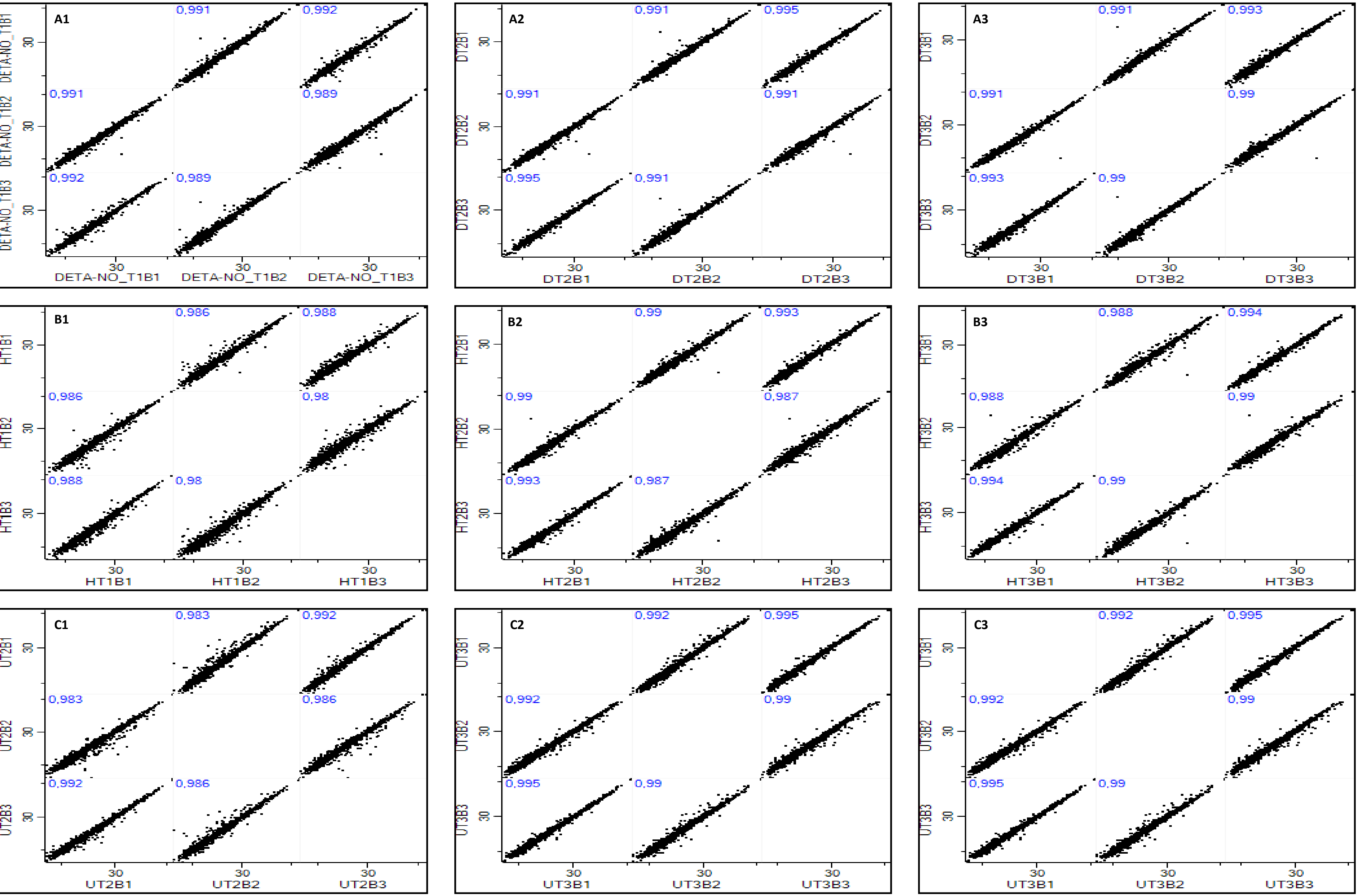

Figure S - 3. The multi-scatter plots using logged LFQ intensity of all quantifiable proteins, were performed between biological replicates of any one treatment condition at one time point. The graphs show a high degree of correlation, with the minimum correlation score above 0.97. Indicating good quantitative reproducibility.

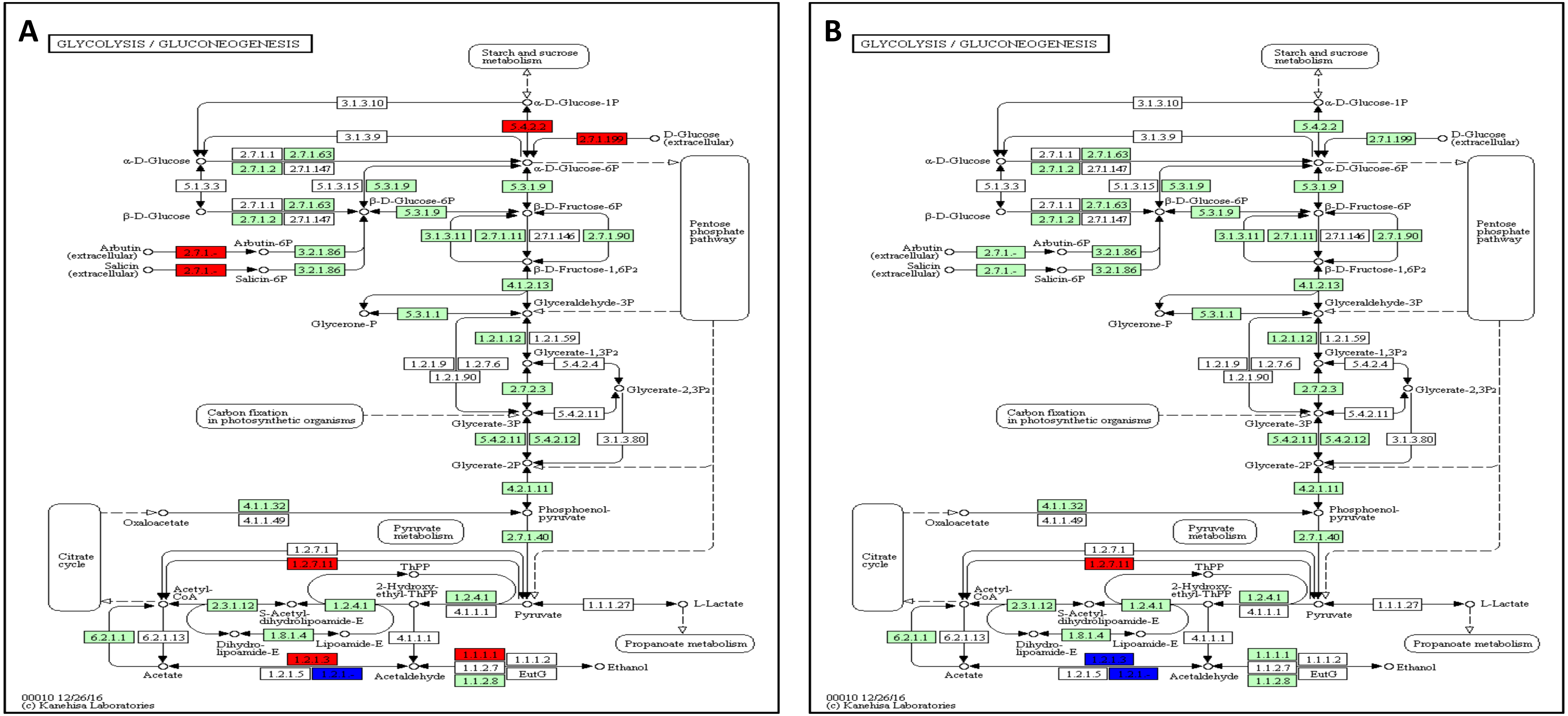

Figure S - 4. A KEGG pathway of Glycolysis/Gluconeogenesis. Red represents proteins with increased abundance; blue represents proteins with decreased abundance. Panel A shows the pathway affected by DETA-NO treatment and panel B shows the pathway affected by H_2O_2 treatment. In panel A; MSMEG_2116 and MSMEG_2117 (both EC number 2.7.1.-), MSMEG_4646 and orB (both EC number 1.2.7.11), pgm (EC number 5.4.2.2), MSMEG_2597 and MSMEG_1543 (both EC number 1.2.1.3), adhE1 and MSMEG_5287 (both EC number 1.1.1.1) are shown. In panel B; MSMEG_1543 (EC number 1.2.1.3), MSMEG_4646 (EC number 1.2.7.3) and MSMEG_6297 (EC number 1.2.-.-) are shown.

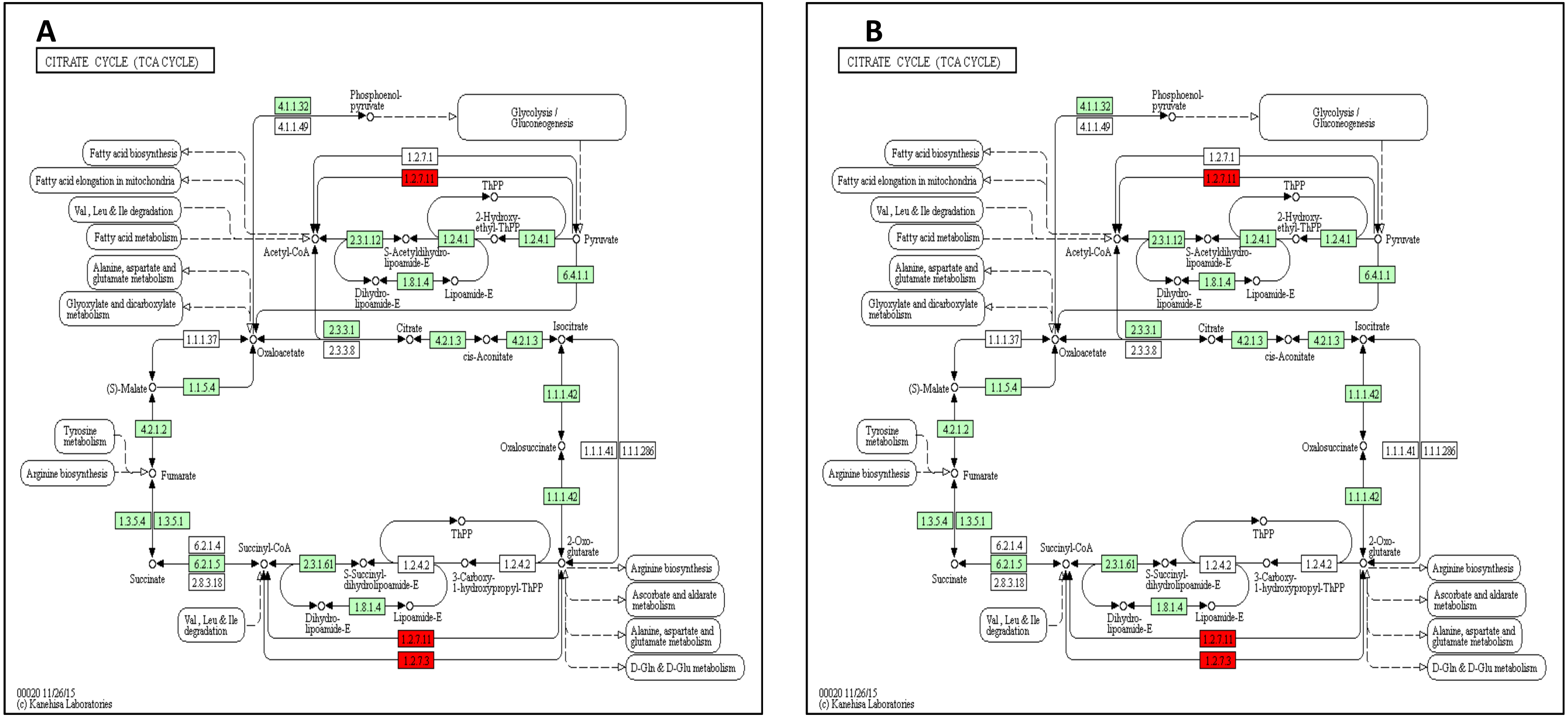

Figure S - 5. A KEGG pathway diagram of the Citrate Cycle. Red represents proteins with increased abundance, blue represents proteins with decreased abundance. Panel A shows the pathway affected by DETA-NO treatment and Panel B shows the pathway affected by H_2O_2 treatment. In panel A and B, MSMEG_4646 and orB are shown with EC numbers 1.2.7.11 and 1.2.7.3.

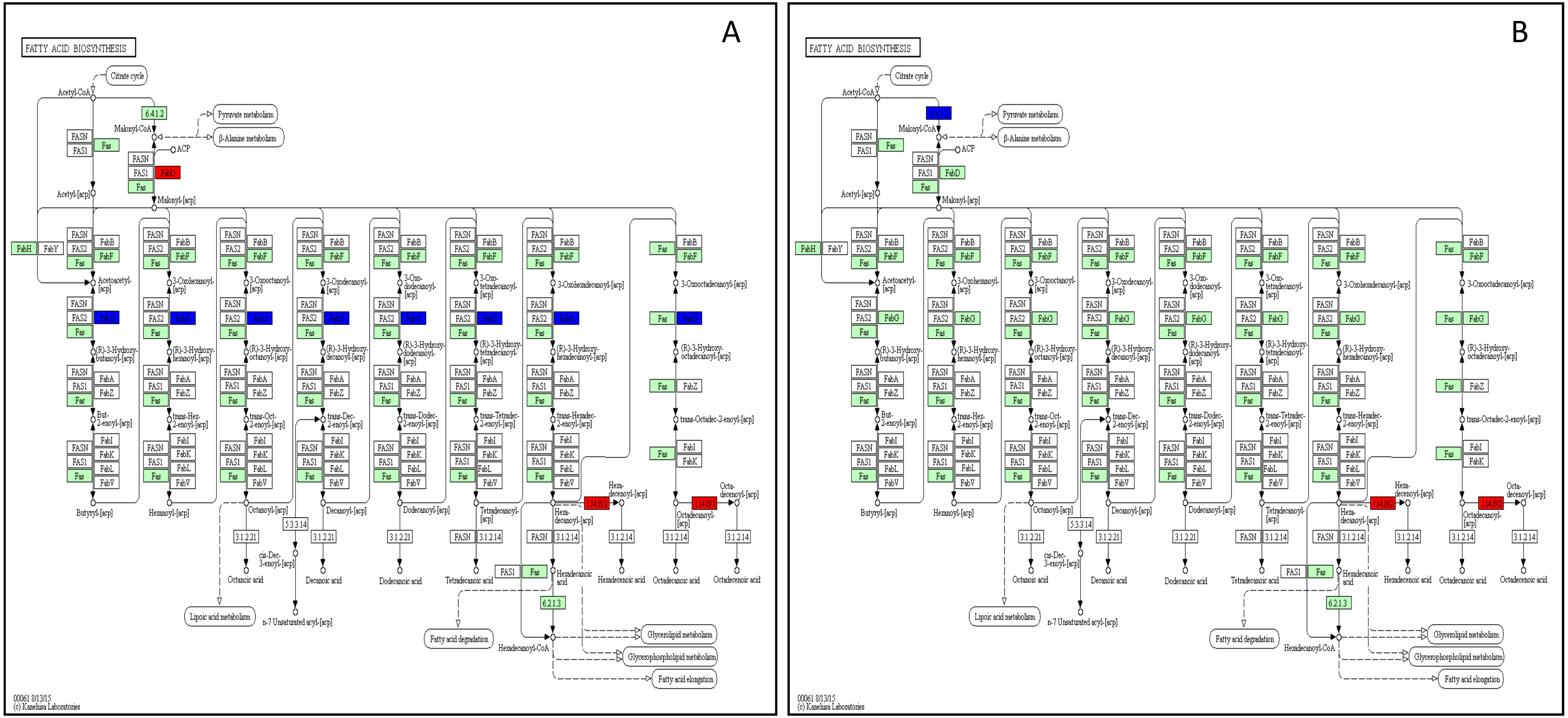

Figure S - 6. A KEGG pathway diagram for Fatty Acid Biosynthesis. in Red represents proteins with increased abundance, blue represents proteins with decreased abundance. Panel A shows the pathway affected by DETA-NO treatment and panel B shows the pathway affected by H_2O_2 treatment. In panel A, des is shown with EC Number 1.14.192, MSMEG_2536 is shown as FabG, and MSMEG_2597 is shown as FabD. In panel B, accD6 is shown as EC number 6.4.1.2 and des is shown as EC Number 1.14.192.

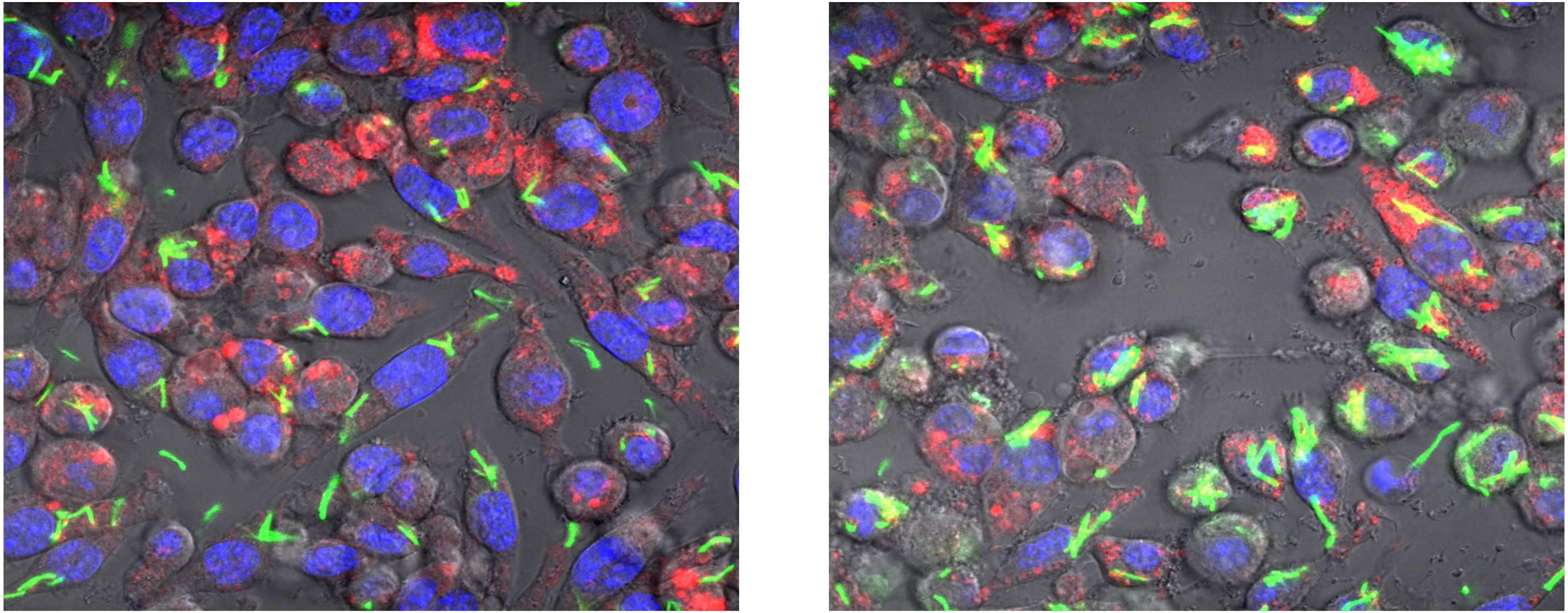

Figure S – 7. RAW 264.7 cells were infected with GFP-labelled *M. smegmatis* at a multiplicity of infection (MOI) of 10:1. The nuclei of RAW cells were visualised with Hoechst and lysosomal compartments with LysoTracker® Red DND-99. Infection of marcophages was followed using a Zeiss Axiovert 200M LSM 510 Meta Confocal Microscope. Panel A – represents an early time point of infection, where most bacteria are intracellular. Panel B – represents a later time point, after bacteria have survived and multiplied intracellularly.
